# Longitudinal Trajectories of Cognitive Control and Theory of Mind in Amnestic and Non-Amnestic Mild Cognitive Impairment: Prospective Associations of Objective Sleep Duration

**DOI:** 10.3390/jcm15155780

**Published:** 2026-07-23

**Authors:** Areti Batzikosta, Magda Tsolaki, Paschalis Steiropoulos, Georgia Papantoniou, Ioanna Giannoula Katsouri, Maria Sofologi, Glykeria Tsentidou, Athanasios Voulgaris, Stave Vergopoulou, Konstantinos I. Bougioukas, Despina Moraitou

**Affiliations:** 1 Laboratory of Psychology, Department of Cognition, Brain and Behavior, School of Psychology, Faculty of Philosophy, Aristotle University of Thessaloniki (AUTH), 54124 Thessaloniki, Greece; gltsentidou@gmail.com (G.T.); mpougioukas@auth.gr (K.I.B.); demorait@psy.auth.gr (D.M.); 2 Laboratory of Neurodegenerative Diseases, Center of Interdisciplinary Research and Innovation (CIRI-AUTH), Balcan Center, Buildings A & B, 57001 Thessaloniki, Greece; tsolakim1@gmail.com (M.T.); gpapanto@uoi.gr (G.P.); 3 Greek Association of Alzheimer’s Disease and Related Disorders (GAADRD), Petrou Sindika 13 Str., 54643 Thessaloniki, Greece; 4 Department of Respiratory Medicine, Medical School, Democritus University of Thrace, 68100 Alexandroupolis, Greece; steiropoulos@yahoo.com (P.S.); thanasisvoul@hotmail.com (A.V.); 5 Laboratory of Psychology, Department of Early Childhood Education, School of Education, University of Ioannina, 45110 Ioannina, Greece; m.sofologi@uoi.gr; 6 Institute of Humanities and Social Sciences, University Research Centre of Ioannina (URCI), 45110 Ioannina, Greece; 7 Department of Occupational Therapy, Faculty of Health and Caring Sciences, University of West Attica, 12243 Athens, Greece; ykatsouri@uniwa.gr; 8 Department of Psychology, School of Health Sciences, Neapolis University Pafos, 8042 Pafos, Cyprus; 9 Department of Arts and Humanities, School of Humanities, Social and Educational Sciences, European University of Cyprus (EUC), 6 Diogenes 2404 Engomi, P.O. Box 22006, 1516 Nicosia, Cyprus; svergopo@gmail.com

**Keywords:** aging, executive functions, non-literal speech comprehension, objective sleep indices, social cognition

## Abstract

**Background/Objectives:** Sleep disturbances are increasingly implicated in age-related cognitive decline and may contribute to heterogeneity in cognitive and social–cognitive functioning in Mild Cognitive Impairment (MCI). Nevertheless, it remains unclear whether longitudinal actigraphy-derived sleep parameters predict social cognition, including theory of mind (ToM), across amnestic (aMCI) and non-amnestic (naMCI) MCI subtypes. This study examined longitudinal trajectories of cognitive control and ToM in individuals with aMCI and naMCI and healthy older adults, and investigated whether actigraphy-derived sleep indices prospectively predict subsequent cognitive control and higher-order social cognition across these groups. **Methods:** A total of 179 participants (46 healthy controls, 75 aMCI, 58 naMCI) were assessed across three waves over approximately 16–20 months. Actigraphy-derived sleep indices were obtained using wrist actigraphy. Cognitive control was assessed with Delis–Kaplan Executive Function System subtests, whereas ToM was evaluated through tasks involving non-literal language comprehension, metaphor and proverb interpretation, higher-order mentalizing, and emotion recognition. Longitudinal trajectories were examined using mixed-design ANOVAs, and prospective sleep effects were tested using longitudinal path models. **Results:** Both cognitive control and ToM declined over time in the MCI groups, whereas healthy controls showed relative stability. Subtype differences in cognitive control emerged primarily in higher-order planning and rule-monitoring processes, with naMCI showing greater impairment. ToM revealed a clearer and more consistent gradient (healthy controls > aMCI > naMCI), particularly in higher-order inferential tasks. Among the actigraphy-derived sleep indices, only TST prospectively predicted later ToM performance, whereas prospective associations with cognitive control were limited and inconsistent. **Conclusions:** Reduced sleep duration was prospectively associated with poorer subsequent performance in higher-order social cognition, independently of the executive-control measures examined. These findings highlight phenotypic heterogeneity across MCI subtypes and suggest that objectively measured sleep duration may serve as a clinically relevant longitudinal marker of social–cognitive vulnerability. Future studies are needed to determine whether interventions targeting sleep duration are associated with improved social–cognitive outcomes.

## 1. Introduction

Sleep is a fundamental biological process essential for maintaining cognitive, emotional, and functional integrity across the lifespan [1]. Rather than a passive state of rest, sleep constitutes an active neurobiological period that supports memory consolidation, synaptic homeostasis, emotional regulation, and large-scale neural network integration [2]. In age-related neurodegeneration, persistent disruptions in sleep continuity and efficiency have been consistently associated with accelerated cognitive decline, highlighting sleep as a clinically relevant and potentially modifiable factor in age-related neurocognitive vulnerability [3,4]. However, the clinical manifestations and underlying mechanisms of sleep disturbances vary considerably across neurodegenerative disorders, reflecting disease-specific neuropathological processes [5]. Systematic sleep assessment has also been recognized as a valuable component of routine clinical evaluation across diverse patient populations, underscoring the broader clinical importance of sleep beyond neurodegenerative disorders [6].

In this context, objective sleep assessment methods such as wrist actigraphy have gained increasing prominence, providing ecologically valid measures of habitual sleep patterns that overcome limitations inherent to self-reported sleep indices [7]. However, despite growing evidence linking sleep disturbances to cognitive decline, longitudinal studies using actigraphy to examine whether objectively measured sleep predicts social cognition, particularly theory of mind (ToM), across mild cognitive impairment (MCI) subtypes remain scarce. Addressing this gap may improve our understanding of the role of sleep in the evolution of cognitive control and social cognition during the prodromal stages of neurodegenerative disease.

Mild cognitive impairment (MCI) is a clinically heterogeneous syndrome characterized by cognitive decline greater than expected for normal aging but insufficient for a dementia diagnosis [8]. Its two primary subtypes, amnestic MCI (aMCI) and non-amnestic MCI (naMCI), exhibit distinct cognitive profiles and underlying neuropathological substrates. aMCI is predominantly characterized by episodic memory impairment and is closely associated with Alzheimer’s disease–related pathology, including early hippocampal dysfunction [9]. Accumulating evidence indicates that individuals with aMCI frequently exhibit sleep disturbances that may interfere with hippocampal-dependent memory consolidation processes, particularly during non-rapid eye movement (NREM) sleep [10]. In contrast, naMCI primarily affects executive and attentional domains and is more commonly associated with vascular and frontotemporal pathologies, implicating dysfunction within prefrontal and frontoparietal control networks [9]. These neurocognitive distinctions suggest that associations between objectively measured sleep and longitudinal cognitive and social–cognitive outcomes may differ across MCI subtypes.

Objective sleep parameters derived from actigraphy including total sleep time (TST), sleep efficiency (SE), and wake after sleep onset (WASO) capture complementary aspects of habitual sleep duration and continuity over extended periods [11]. Reduced TST and SE, along with increased WASO and fragmentation, have been associated with impairments across multiple cognitive domains, including executive functioning, attention, and processing speed [12,13]. Although actigraphy does not provide information on specific sleep stages or sleep architecture, it offers robust indices of habitual sleep duration and continuity in real-world settings. Accordingly, the present study focuses on longitudinal behavioral associations between objectively measured sleep patterns, cognitive and social–cognitive outcomes rather than on stage-specific sleep mechanisms. From a systems-level perspective, sustained reductions in total sleep time and disruptions in sleep continuity may be associated with cumulative vulnerability of large-scale neural networks, particularly prefrontal control systems, rather than alterations in discrete sleep architecture, thereby plausibly impacting executive-dependent cognitive and social–cognitive functions. Importantly, emerging evidence suggests that sleep alterations may be associated with MCI underlying pathology, potentially contributing to heterogeneity in cognitive trajectories in these patients [14,15].

Beyond traditional cognitive domains, increasing attention has focused on social cognition, particularly ToM, defined as the ability to infer and interpret others’ beliefs, intentions, and communicative meanings [16]. ToM is central to effective social interaction and everyday functioning; however, behavioral tasks used to assess higher-order ToM in older adults often also place demands on pragmatic language processing, contextual integration, and executive control, including inhibitory control, working memory, abstraction, and the inhibition of literal interpretations [17,18]. Contemporary models conceptualize ToM as a multilevel construct, ranging from first-order mental state attribution to higher-order and metarepresentational processes that require simultaneous integration of multiple perspectives and the interpretation of non-literal speech, metaphors, and proverbs [19,20]. ToM is also closely related to the broader constructs of mentalizing and metacognition. Although these constructs partially overlap, ToM primarily concerns the attribution of others’ mental states, whereas metacognition refers to the monitoring and regulation of one’s own cognitive processes [21]. Consequently, higher-order ToM tasks engage not only social–cognitive reasoning but also self–other representational processes and executive resources required for flexible interpretation of complex social information [22].

Evidence indicates that MCI is associated with impairments not only in core cognitive abilities but also in social–cognitive functioning. The clinical relevance of these deficits extends beyond neuropsychological performance, as impaired social cognition has been associated with poorer interpersonal functioning, reduced social participation, impaired everyday decision-making, diminished quality of life, and increased caregiver burden [23]. Difficulties in emotion recognition, intention attribution, and higher-order ToM abilities have been documented in both aMCI and naMCI and are thought to contribute to these functional difficulties by compromising communication, social adaptation, and functional independence [24].

Neurobiologically, ToM relies on distributed networks encompassing the medial prefrontal cortex, temporoparietal junction, anterior cingulate cortex, and limbic structures, which substantially overlap with networks supporting executive control and emotional regulation [25]. Given that sleep disturbances have been associated with alterations in prefrontal executive functioning and affective regulation, overlap between these neural systems may contribute to the observed longitudinal associations between sleep and ToM performance, although the underlying mechanisms remain to be established [26,27]. Beyond alterations in functional brain networks, accumulating evidence suggests that sleep disturbances may also contribute to cognitive deterioration through impaired glymphatic clearance of neurotoxic metabolites, increased neuroinflammatory activity, synaptic dysfunction, and the accumulation of neurodegeneration-related proteins. These pathophysiological mechanisms may further increase the vulnerability of neural systems supporting higher-order cognitive and social–cognitive functioning [28]. Nevertheless, empirical evidence directly examining the longitudinal relationship between objectively measured sleep and ToM performance in MCI remains limited.

At a systems level, sleep facilitates both local synaptic regulation and functional integration across large-scale neural networks. Disruptions in sleep continuity and efficiency may therefore be associated with cumulative vulnerability across cognitive and social–cognitive domains, particularly in individuals with pre-existing neurocognitive compromise such as MCI [1,29]. Although the present study does not incorporate neuroimaging, molecular biomarkers or direct measures of sleep architecture, examining longitudinal associations between objectively measured sleep parameters and behavioral outcomes offers valuable clinical insights and complements ongoing neurobiological research. Actigraphy-based assessment represents a feasible and scalable approach for identifying early behavioral markers of cognitive and social–cognitive decline in both clinical and community settings [30,31].

Despite growing recognition of the associations among sleep disturbances, cognitive decline, and social cognition, several important gaps remain in the literature. Most previous studies have focused on memory performance, global cognition, or cross-sectional associations, whereas considerably less attention has been devoted to higher-order social–cognitive abilities such as ToM. Furthermore, studies employing objective sleep assessment methods, particularly actigraphy, remain relatively scarce in MCI populations. Importantly, to our knowledge, no previous longitudinal study has examined whether actigraphy-derived sleep indices prospectively predict higher-order social cognition ToM across aMCI and naMCI subtypes while simultaneously characterizing longitudinal cognitive-control trajectories. This represents an important gap in understanding behavioral markers of social–cognitive vulnerability in MCI.

Hence, the primary aims of the present longitudinal study were to characterize longitudinal trajectories of cognitive control and higher-order social cognition in individuals with aMCI and naMCI compared with cognitively healthy older adults, and to determine whether actigraphy-derived sleep indices prospectively predicted these outcomes. By examining these relationships across three assessment waves over approximately 16–20 months, the study sought to address the current lack of longitudinal evidence regarding the role of objectively measured habitual sleep in cognitive and social–cognitive functioning across MCI subtypes.

Hypotheses of the Study

Based on existing evidence from sleep physiology, cognitive neuroscience, and social cognition research, the following hypotheses were tested:

1. Cognitive control and ToM performance were expected to decline across the three assessment waves. Individuals with naMCI were expected to demonstrate poorer performance than aMCI patients, while healthy controls were expected to show the highest performance compared to both MCI groups.
2. Actigraphy-derived sleep indices, including TST, SE, and WASO, were expected to show prospective associations with subsequent performance in cognitive-control and ToM tasks across assessment waves.

## 2. Materials and Methods

### 2.1. Design

The study comprised three assessment waves (T1, T2, and T3) conducted over approximately 16–20 months. Following the baseline evaluation, participants completed two subsequent assessments at comparable intervals, with the second assessment (T2) taking place approximately 8–10 months after baseline and the third assessment (T3) approximately 16–20 months after baseline. All assessment waves adhered to an identical, standardized protocol, incorporating objective and subjective sleep measures. Cognitive-control functioning and specific ToM aspects were assessed at each wave using validated assessment tools, which are described in detail in the sections below.

It should be noted that data from the first wave have contributed to previous publications addressing cross-sectional associations between sleep parameters and specific cognitive and/or social–cognitive outcomes [32,33]. In addition, sleep data from all three assessment waves were previously reported in a publication regarding objective and subjective sleep trajectories [14] in MCI. The present study does not reproduce any previously published analyses but extends previous work by characterizing longitudinal trajectories of cognitive control and ToM across three assessment waves and by examining whether actigraphy-derived sleep indices prospectively predict these longitudinal cognitive and social–cognitive outcomes in individuals with aMCI and naMCI compared with healthy older adults.

### 2.2. Participants

An a priori power analysis using G*Power version 3.1.9.7 [34] indicated that a minimum sample of 148 participants would be required to achieve 80% statistical power. Accordingly, 185 participants were initially recruited and underwent comprehensive assessments. Six participants were excluded based on predefined eligibility criteria to enhance sample homogeneity and minimize confounding factors: one cognitively healthy participant with possible subjective cognitive impairment (SCI), characterized by persistent self-reported cognitive complaints; one participant diagnosed with early-stage dementia; and four participants receiving pharmacological treatment for sleep disorders. These exclusions were made in consultation with the principal investigator and the research team. Consequently, the final sample comprised N = 179 individuals, 46 cognitively healthy controls, 75 patients diagnosed with aMCI, and 58 with naMCI. The sample included 53 men and 126 women, with a mean age of 70.23 years (SD = 4.74) and a mean educational attainment of 12.35 years (SD = 3.22) (Table 1).

**Figure 1:**
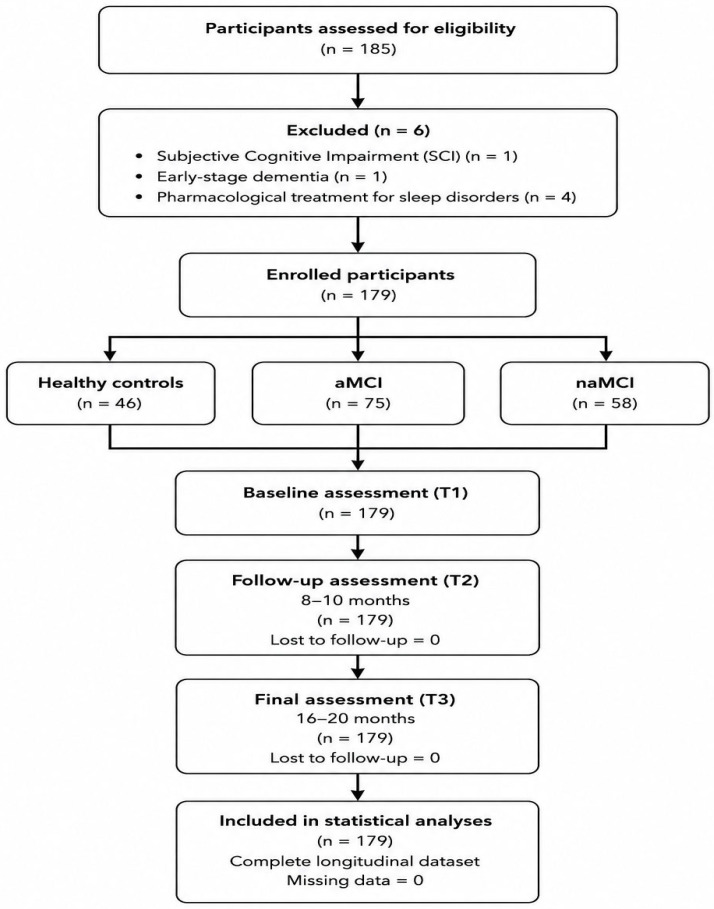
Flow diagram of participants formally assessed for study eligibility recruitment, eligibility assessment, follow-up, and inclusion in the statistical analyses.

Diagnostic status was reviewed prior to each assessment wave based on the participants regularly updated clinical records maintained by the Greek Association of Alzheimer’s Disease and Related Disorders. No participant demonstrated diagnostic conversion from MCI to dementia or transition between the amnestic and non-amnestic MCI subtypes during the 16–20-month follow-up period. Participants were recruited and followed through the Day Care Centers of the Greek Association of Alzheimer’s Disease and Related Disorders, where they routinely attended scheduled follow-up assessments as part of their standard clinical care. Consequently, no participants were lost to follow-up, and complete longitudinal data were available for all individuals included in the study. A participant flow diagram summarizing recruitment, eligibility, follow-up, and inclusion in the statistical analyses is provided in Figure 1.

### 2.3. Inclusion Criteria

Participants were eligible if they were aged 65 years or older, were native speakers of Greek and had completed a minimum of six years of formal education. Following a comprehensive neuropsychological evaluation conducted in the Day Care Centers of the Greek Association of Alzheimer’s Disease and Related Disorders (GAADRD), participants were categorized into three groups: cognitively healthy controls, individuals with aMCI, and those with naMCI. All participants were recruited into the present study only after they had received an established clinical diagnosis through the standardized diagnostic procedures of the GAADRD. Thus, the research team did not perform the diagnostic classification but enrolled participants with pre-existing clinical diagnoses assigned by the Centers’ multidisciplinary clinical team. The classification adhered to Petersen’s diagnostic criteria [35] and the *Diagnostic and Statistical Manual of Mental Disorders, Fifth Edition, Text Revision* (DSM-5-TR) [36], supplemented by clinical and biological data, including MRI scans and cerebrospinal fluid (CSF) biomarkers suggestive of neurodegenerative pathology for all participants. It should be noted that neuroimaging and CSF data were not directly accessible to us, as these assessments were performed by the Greek Association of Alzheimer’s Disease Centers within their diagnostic procedures prior to participant inclusion.

The control group consisted of 46 cognitively healthy community-dwelling adults. All participants demonstrated preserved cognitive function, as evidenced by Montreal Cognitive Assessment (MoCA) scores ranging from 27 to 30 (M = 27.8, SD = 0.74), consistent with their clinical classification as cognitively healthy.

The aMCI group consisted of 75 individuals diagnosed with MCI within the previous two years. Diagnoses were made in accordance with DSM-5-TR criteria for mild neurocognitive disorders [36] and were supported by comprehensive neuropsychological testing, neurological examinations, neuroimaging, and psychiatric assessments. Inclusion criteria required (a) a confirmed diagnosis of mild neurocognitive disorder and (b) performance at least 1.5 standard deviations below normative means on an episodic memory task. All participants in this group were classified as having the amnestic subtype, with MoCA scores ranging from 24 to 29 (M = 24.59, SD = 2.7).

The naMCI group comprised 58 individuals diagnosed with MCI within the previous two years. Inclusion required (a) a clinical diagnosis of mild neurocognitive disorder according to DSM-5-TR criteria [36] and (b) performance at least 1.5 standard deviations below normative means in one or more cognitive domains other than memory. Following evaluation, all participants were classified as having naMCI, with MoCA scores ranging from 20 to 29 (M = 26.10, SD = 2.47).

### 2.4. Exclusion Criteria

Participants were excluded if they met any of the following criteria: (a) history of psychiatric disorders, (b) substance use or alcohol dependence, (c) prior traumatic brain injury, (d) history of neurological disorders (e.g., stroke, Parkinson’s disease, epilepsy, multiple sclerosis, or other neurological conditions affecting cognition) (e) diagnosed sleep disorders, (f) current use of medications for sleep-related issues, (g) use of antidepressants or anxiolytics, or (h) presence of cognitive complaints and/or a diagnosis of MCI within the healthy control group.

All participants underwent a comprehensive neuropsychological evaluation by multidisciplinary clinical team of the centers to establish diagnostic status. Mood and affective symptoms were assessed using the Geriatric Depression Scale [37,38], Beck Depression Inventory [39], Beck Anxiety Inventory [40], and Short Anxiety Screening [41,42]. Neuropsychiatric symptoms were evaluated via the Neuropsychiatric Inventory [43,44].

Global cognitive functioning was measured with the Mini-Mental State Examination (MMSE) [45,46] and the MoCA [47,48]. Executive functions were further evaluated using the Functional Cognitive Assessment Scale [49], which includes tasks simulating activities of daily living, complemented by a battery of standardized tests assessing memory, attention, executive function, and language abilities. The Global Deterioration Scale (GDS) [50] was employed to rate the severity of cognitive decline, with stage 1 indicating no decline and stage 3 corresponding to MCI. For a detailed description of the neuropsychological instruments, refer to Tsolaki et al. [51].

Baseline comparisons revealed no significant group differences in age, F (2, 176) = 2.977, *p* = 0.054; years of education, F (2, 176) = 0.452, *p* = 0.637; or gender distribution, χ^2^ = 1.849, *p* = 0.397.

### 2.5. Ethics

All participants received detailed information about the study’s objectives through both verbal explanations and written materials, with explicit assurances regarding the confidentiality of their personal data. Written informed consent was obtained prior to the baseline assessment, confirming voluntary participation and emphasizing that participants could withdraw at any time without penalty. Demographic data, including age, gender, and educational attainment, were collected in compliance with European Union regulations under the General Data Protection Regulation (GDPR, effective 28 May 2018), which allows the use of sensitive personal information for research purposes. Participants were also informed of their right to request the removal of their data from the study database. The study protocol was approved by the Scientific and Ethics Committee of the Greek Association of Alzheimer’s Disease and Related Disorders (approval code: 29/15-02-2017) and conducted in accordance with the ethical standards of the Declaration of Helsinki.

### 2.6. Procedure

Participant recruitment was conducted by undergraduate psychology interns at the Day Care Centers of the GAADRD. Their role was limited to contacting eligible participants by telephone to invite them to participate in the study. Individuals who agreed to participate were subsequently contacted by the study psychologist. The study psychologist provided comprehensive information regarding the study’s aims and procedures. All study procedures, including informed consent and neuropsychological assessment, were conducted exclusively by the study psychologist. Participants then scheduled two morning sessions within one week, each lasting up to one hour, to complete the required assessments.

Due to the longitudinal design, participants were re-contacted for follow-up assessments at two additional time points. Each follow-up session followed the same standardized assessment protocol to maintain consistency across assessments waves. The same D-KEFS tasks were administered at all three assessment waves. In contrast, the ToM assessment employed three comparable sub-batteries of Natsopoulos’ ToM battery, each comprising different tasks assessing the same ToM domains, in order to minimize potential practice and carryover effects across repeated assessments. Similarly, the dynamic emotion-recognition task (TASIT—Part I) was divided into three comparable subsets that were administered across the three assessment waves. Assessments were conducted individually in a quiet, comfortable setting. Participants did not receive any compensation for their involvement.

### 2.7. Objective Assessment of Sleep

Actigraphy is a non-invasive method used to assess sleep–wake patterns through the analysis of movement data [52]. It is extensively employed in research settings due to its capacity to monitor activity continuously in naturalistic, real-world environments. In this study, all participants wore the Actiwatch Spectrum Pro (Philips Respironics, Murrysville, PA, USA) continuously on the non-dominant wrist for seven consecutive days and nights. Sleep data were analyzed using Actiware software, version 5.57.0006 (Philips Respironics, Murrysville, PA, USA). The wrist-worn device records movement and ambient light exposure, providing objective information on activity levels and sleep–wake patterns. Unlike polysomnography, actigraphy relies on accelerometer-based motion detection rather than electrophysiological signals, offering indirect yet reliable estimates of sleep–wake behavior. It is widely used in sleep medicine to evaluate sleep disorders, circadian rhythm disturbances, and daily activity patterns [53].

Sleep data were processed using the accompanying Actiware software. Rest intervals, including bedtime and final wake time, were identified automatically by the software based on participants’ activity patterns. Sleep–wake scoring was performed using the software’s standard automated scoring procedure with medium wake-threshold sensitivity. Participants were also provided with sleep diaries to record bedtime, wake time, and periods during which the device was removed. However, because diary completion was inconsistent, diary data were not incorporated into the scoring procedure. Only the primary nocturnal sleep interval identified by the software was included in the analyses; daytime naps were not analyzed. Total sleep time (TST), wake after sleep onset (WASO), and sleep efficiency (SE) were automatically calculated and visualized by the Actiware software, facilitating data review [54]. Mean values across the seven-night recording period, including both weekdays and weekends, were used for the statistical analyses.

### 2.8. Cognitive Measures

In line with the existing literature highlighting executive function deficits in individuals with MCI, we employed a battery of tests to assess core cognitive-control processes, including inhibitory control, set-shifting, cognitive flexibility, and planning. These domains were evaluated using the following tests from the Delis–Kaplan Executive Function System (D-KEFS) [55], namely the Verbal Fluency Test, Standard Form (D-KEFS VF, SF), the Color–Word Interference Test, Standard Form (D-KEFS C-WIT, SF), and the Tower Test, Standard Form (D-KEFS TT, SF) [55]. These tests were selected due to their established sensitivity to executive dysfunction in MCI populations and their relevance to the cognitive-control processes examined in the present study [56].

The Verbal Fluency Test includes phonemic fluency (Condition I), semantic/category fluency (Condition II), category switching (Condition IIIa), and switching accuracy (Condition IIIb), enabling assessment of lexical retrieval, semantic access, and cognitive flexibility, respectively. Although the phonemic and basic semantic fluency conditions were administered as part of the standard D-KEFS protocol, they were not included in the primary analyses because they predominantly assess lexical retrieval and semantic access rather than the cognitive-control processes that constituted the focus of the present study. Given the study’s emphasis on cognitive control and executive dysfunction in MCI, analyses focused specifically on the category switching condition, which more directly indexes cognitive flexibility and set-shifting abilities. Specifically, two variables of interest were derived from the Verbal Fluency Test: (a) total correct responses in the category switching condition (Condition IIIa) of the semantic fluency subtest as well as (b) switching accuracy (Condition IIIb), indexing cognitive flexibility.

Two variables of interest were derived from the Color–Word Interference Test: (a) number of uncorrected errors in the inhibition condition (Condition III), and (b) number of uncorrected errors in the inhibition/switching condition (Condition IV), reflecting inhibitory control and cognitive flexibility, respectively. Error-based indices were selected as more sensitive indicators of inhibitory control and cognitive flexibility deficits in clinical populations [57,58].

Finally, three variables reflecting higher-order planning and rule-monitoring processes were derived from the Tower Test: (a) total achievement score, (b) total number of rule violations, and (c) total number of problems administered, indexing planning and problem-solving ability. Higher achievement scores indicated better performance, whereas higher rule violations reflected poorer performance.

Detailed administration and scoring procedures have been reported previously [32]; therefore, only the measures relevant to the present longitudinal analyses are summarized here.

### 2.9. Theory of Mind Assessment

ToM abilities were assessed using selected components of Natsopoulos’ ToM battery [59,60,61], a comprehensive multidimensional assessment comprising multiple tasks that evaluate both traditional and higher-order ToM aspects, complemented by a dynamic emotion recognition task (TASIT—Part I) [62]. Compared with conventional ToM measures, the battery includes a broader range of social–cognitive processes, including indirect speech understanding, metaphor and proverb comprehension, faux pas understanding, and higher-order mental state reasoning. For the longitudinal phase of the study, the battery was organized into three comparable sub-batteries, each including different but comparable tasks assessing the same ToM domains, to minimize potential order and practice effects across repeated assessments. The individual tasks were developed for the Greek cultural context based on internationally established ToM paradigms, whereas TASIT—Part I has been shown to be suitable for cross-cultural administration without the use of voice [62,63]. Natsopoulos’ ToM battery is currently undergoing psychometric evaluation by our research group at the Aristotle University of Thessaloniki. Although the psychometric validation of the complete battery has not yet been completed, several individual tasks have already undergone psychometric evaluation, demonstrating satisfactory structural validity through confirmatory factor analysis and, where applicable, acceptable internal consistency [60,61]. The remaining tasks are currently being examined using the same psychometric procedures, including confirmatory factor analysis and internal consistency estimation.

The original version of the non-literal speech task comprised 12 short vignettes designed to display humorous, ironic, sarcastic, and faux pas social interactions (three vignettes per condition), together with corresponding control stories. For the longitudinal study, the original material was divided into three comparable sub-batteries, each comprising one humor, one irony, one sarcasm, and one faux pas vignette, together with the corresponding control stories, to minimize potential order and practice effects across repeated assessments. Two variables were derived from the non-literal speech tasks (humor, irony, sarcasm, and faux pas stories): (a) recognition that a statement departs from its literal meaning or is socially inappropriate (non-literal speech 1; score range: 0–4 per assessment wave) and (b) understanding of the speaker’s communicative intention underlying the non-literal meaning (non-literal speech 2; score range: 0–11 per assessment wave). Specifically, for the humor, irony, and sarcasm stories, communicative intention was scored from 0 to 2 points per story, whereas for the faux pas story, understanding of the speaker’s intention, the appropriateness of the remark, and its social–emotional consequences was assessed using five scored questions (score range: 0–5). Control questions were administered to ensure adequate story comprehension but were not included in the scoring.

The original version of the Proverbs in Context task comprised 12 proverbs presented in context, each accompanied by four interpretation options. For the longitudinal study, the material was divided into three comparable sets of four proverbs, with one set administered at each assessment wave. One option represented a literal meaning (0 points), one an irrelevant interpretation (0 points), and two reflected the intended figurative meaning, receiving 1 and 2 points, respectively, according to the accuracy and completeness of the interpretation. Participants were required to select the most accurate interpretation of each proverb. The total score ranged from 0 to 8 per assessment wave, with higher scores indicating better ability to interpret abstract, non-literal meaning. One variable was derived from this task, namely the total score reflecting proverb comprehension.

The Verbal Metaphors in Context task was originally developed with 12 sentences containing metaphorical verbs. For the purposes of the longitudinal study, the material was divided into three comparable sets of four items, with one set administered at each assessment wave. Each sentence was accompanied by three alternative sentences rephrasing the original. Participants were required to select the sentence that best captured the meaning of the original sentence. Only the correct response received 1 point, yielding a total score ranging from 0 to 4 per assessment wave. One variable was derived from this task, namely the total number of correct responses, indexing verbal metaphor comprehension.

The Nominal Metaphors in Context task included 12 sentences containing nominal metaphors. To accommodate the longitudinal design, the material was organized into three comparable sets of four items, with one set administered at each assessment wave. Each sentence was accompanied by three alternative interpretations rephrasing the original sentence. Participants were required to select the option that best conveyed the meaning of the original sentence. Only the correct response received 1 point, yielding a total score ranging from 0 to 4 per assessment wave. One variable was derived from this task, namely the total number of correct responses, indexing nominal metaphor comprehension.

The Third-Order ToM task consisted of three comparable “double bluff” stories, with one story administered at each assessment wave. This task evaluates advanced ToM reasoning by requiring participants to infer one character’s beliefs about another character’s beliefs and intentions. Participants first answered control questions to ensure comprehension of the story before completing the scored questions assessing higher-order mental state reasoning. Correct responses yielded a total score ranging from 0 to 3 per assessment wave. One variable was derived from this task, namely the total score reflecting the ability to infer higher-order mental states (i.e., beliefs about others’ beliefs and intentions).

The TASIT—Part I: Emotion Evaluation Test (EET) [62] was administered using three comparable forms, with one form presented at each assessment wave to minimize potential order and practice effects. Each form consisted of nine short video clips (15–60 s) depicting everyday social interactions in which professional actors dynamically portrayed six fundamental emotions (happiness, surprise, sadness, anger, anxiety, and disgust) or a neutral emotional expression. The videos were presented without sound to focus on participants’ ability to interpret dynamic visual cues from facial expressions and gestures. Participants identified the emotion expressed by the actor from seven response options. Three variables were derived from this test: recognition accuracy for neutral emotional expressions (control condition; score range: 0–2 per assessment wave), recognition accuracy for happy emotional expressions (score range: 0–2 per assessment wave), and recognition accuracy for the remaining emotional expressions (sadness, anger, anxiety, disgust, and surprise; score range: 0–6 per assessment wave) based on the total number of correct responses within each category. This operationalization was based on the Greek adaptation of the test, the structural validity of which was examined through confirmatory factor analysis (CFA) [63]. Specifically, the final CFA model supported one latent factor reflecting the decoding of uncertain emotional displays (i.e., sadness, anger, anxiety, disgust, and surprise), whereas recognition of neutral and happy emotional expressions emerged as two distinct observed variables allowed to covary with this factor [63].

All responses were scored offline by the study psychologist according to standardized scoring criteria under the supervision of the senior investigator.

### 2.10. Statistical Analysis

Statistical analyses were conducted using IBM SPSS Statistics version 29.0 [64] and Jamovi version 2.6 [65]. Descriptive statistics were calculated for demographic, sleep, cognitive-control, and ToM variables.

Longitudinal changes in cognitive-control and ToM variables across the three assessment waves (T1, T2, T3) were examined using 3 (Group: HCs, aMCI, naMCI) × 3 (Time: T1, T2, T3) mixed-design ANOVAs, with group as the between-subjects factor and time as the within-subject factor. When significant effects were observed, Scheffé-adjusted post hoc tests were conducted for between-group comparisons and Bonferroni-adjusted pairwise comparisons for within-subject effects across time. When the assumption of sphericity was violated, Greenhouse–Geisser corrections were applied. Effect sizes are reported as partial eta squared (η^2^).

In addition, to control the family-wise Type I error arising from the multiple cognitive-control and ToM outcome variables, Bonferroni-adjusted significance thresholds were applied separately within each outcome family (cognitive control and ToM). A *p*-value < 0.007 was considered statistically significant for the cognitive-control measures (i.e., significant *p* = 0.05/7 dependent variables = 0.007), whereas a *p*-value < 0.006 was considered statistically significant for the ToM measures (i.e., significant *p* = 0.05/9 dependent variables = 0.006).

To examine prospective associations between actigraphy-derived sleep indices and longitudinal cognitive-control and ToM outcomes, longitudinal path models were estimated using structural equation modeling (SEM) in Jamovi, implemented through the lavaan framework. Separate models were estimated for each actigraphy-derived sleep index (TST, SE, and WASO) and each cognitive-control and ToM outcome. Model parameters were estimated using maximum likelihood estimation.

Model fit was evaluated using commonly recommended goodness-of-fit indices, including the chi-squared statistic (χ^2^), the standardized root mean square residual (SRMR), the comparative fit index (CFI), and the root mean square error of approximation (RMSEA). A non-significant chi-squared value (*p* > 0.05) indicates good correspondence between the proposed model and the observed data. However, because the chi-squared statistic is sensitive to sample size and minor model misspecifications, model fit was primarily evaluated using the complementary fit indices (RMSEA, SRMR, and CFI) rather than the chi-squared statistic alone [66,67].

RMSEA values ≤ 0.05 indicate close model fit, values between 0.05 and 0.08 indicate reasonable fit, values between 0.08 and 0.10 indicate mediocre fit, and values > 0.10 indicate poor fit [66,67,68]. SRMR values below 0.05 indicate good model fit, whereas values between 0.05 and 0.08 are considered acceptable [68]. For the CFI, values greater than 0.90 indicate acceptable fit, while values approaching 1.00 reflect excellent model fit [66,67]. Standardized regression coefficients (β) are reported for all estimated structural paths.

## 3. Results

### 3.1. Cognitive-Control Performance

A series of 3 (group) × 3 (time) mixed-design ANOVAs revealed significant main effects of time across all cognitive-control outcomes, indicating longitudinal changes in performance across the three assessment waves. After Bonferroni correction for the cognitive-control outcome family, significant group effects were observed for all measures except CWIT Conditions III and IV, whereas significant time × group interactions were identified for all cognitive-control variables, indicating progressive decline over time and differential longitudinal trajectories across diagnostic groups (Table 2). Similar longitudinal patterns were observed across planning, verbal fluency, and inhibitory control measures (Table 2), although the magnitude of group effects varied across outcomes.

The mean scores and standard deviations across the three assessment points further illustrate distinct group trajectories (Table 3). While healthy controls showed stable performance or slight improvements, both MCI groups exhibited progressive decline across all cognitive-control measures.

Post hoc comparisons consistently indicated that healthy controls outperformed both MCI groups across most measures. Differences between aMCI and naMCI were generally non-significant, apart from planning and rule monitoring processes, where the naMCI group showed greater impairment. In addition, Bonferroni-adjusted comparisons revealed a systematic deterioration in performance across successive assessments (T1 > T2 > T3) (Table 4).

**Figure 2:**
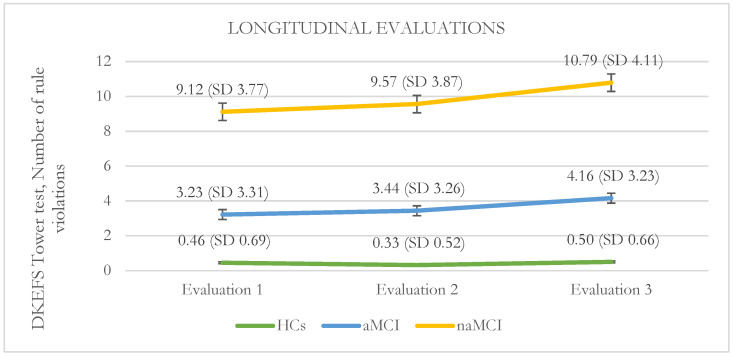
Number of rule violations on the D-KEFS Tower Test across the three assessments by diagnostic group (HCs, aMCI, naMCI).

Notably, the strongest group differences were observed in Tower Test rule violations and total number of problems solved, as these variables clearly distinguished all three groups across time, with healthy controls showing the best performance followed by the aMCI group, whereas the naMCI group exhibited the highest number of rule violations. In particular, the naMCI group demonstrated markedly higher rule violation rates already at baseline, followed by further deterioration over time compared to both aMCI and healthy controls. By contrast, semantic category switching and switching accuracy in the Verbal Fluency Test (Conditions IIIa and IIIb) primarily differentiated healthy controls from both MCI groups, whereas the group effect for the Color–Word Interference Test (Condition III) did not remain statistically significant after applying the Bonferroni-adjusted significance threshold, and differences between aMCI and naMCI remained generally non-significant. Finally, inhibition/switching performance (CWIT Condition IV) showed longitudinal decline across assessments, but limited discrimination between diagnostic groups (see Figure 2).

### 3.2. Theory of Mind Measures

A series of 3 (group) × 3 (time) mixed-design ANOVAs revealed significant main effects of time and group across all ToM variables, indicating longitudinal changes in performance and systematic differences among the three diagnostic groups. Significant time × group interactions were observed for the most ToM measures, reflecting differential longitudinal trajectories across groups. The interaction was not significant for third-order ToM stories and did not survive the Bonferroni-adjusted significance threshold for TASIT—Part I (Neutral) (Table 5).

Overall, healthy controls consistently outperformed both MCI groups across most ToM measures, followed by the aMCI group and in most cases the naMCI group. A clear performance gradient (HC > aMCI > naMCI) was observed for non-literal speech comprehension, metaphor comprehension, proverb interpretation, and third-order mentalizing. Similar patterns were observed for the TASIT emotion recognition measures, although differences between the two MCI groups were not consistently significant.

Across assessment waves, performance declined significantly from T1 to T3 for most ToM measures. Mixed-design ANOVA results for all ToM variables are summarized in Table 5. Mixed-design ANOVA results are summarized in Table 5, and descriptive statistics are presented in Table 6.

Post hoc comparisons consistently showed that healthy controls outperformed both MCI groups across most ToM measures, whereas differences between the aMCI and naMCI groups varied across tasks. Specifically, a clear gradient (HC > aMCI > naMCI) was observed for non-literal speech comprehension, verbal metaphor comprehension, proverb interpretation, and third-order mentalizing.

In addition, the pairwise comparisons revealed significant longitudinal declines across most ToM variables, particularly between T1 and T3 assessments. However, the magnitude and pattern of decline varied across tasks and measures. Detailed results for all ToM measures, including group differences and longitudinal changes, are presented in Table 7.

**Figure 3:**
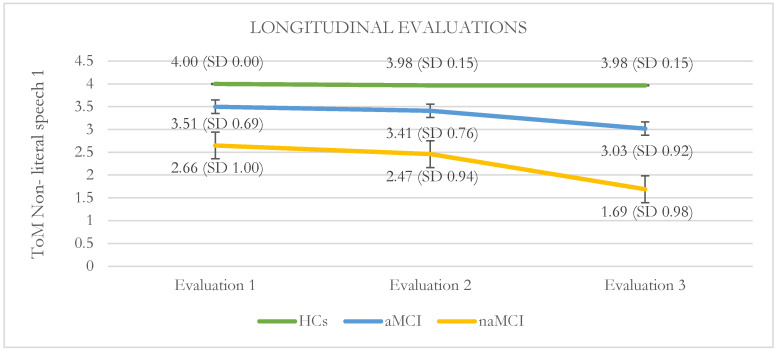
Non-literal speech 1 performance across the three assessments by diagnostic group (HCs, aMCI, naMCI).

Figure 3 illustrates longitudinal changes in non-literal speech 1 performance across the three assessment waves.

The strongest group differences were observed for higher-order ToM measures, particularly non-literal speech 1, non-literal speech 2, and Verbal Metaphors in Context, where the naMCI group consistently obtained lower scores than both the aMCI group and healthy controls across the three assessment waves (Figure 2).

### 3.3. Descriptive Statistics of Objective Sleep Parameters

To facilitate interpretation of the longitudinal path models, descriptive statistics for the actigraphy-derived sleep indices are presented in Table 8. The table summarizes the means and standard deviations of TST, SE, and WASO for each diagnostic group across the three assessment waves. As shown in Table 8, the naMCI group showed lower mean TST and SE, and higher mean WASO, particularly at T3, compared with the other groups. The aMCI group showed intermediate mean values, whereas healthy controls generally showed higher TST and SE and lower WASO across assessment waves.

### 3.4. Longitudinal Path Models of Actigraphy-Derived Sleep Indices and Subsequent Cognitive Control and Theory of Mind Performance

Prior to the longitudinal path analyses, bivariate correlations among the actigraphy-derived sleep indices, cognitive-control measures, and ToM variables across the three assessment waves were examined. The complete correlation matrices are presented in Supplementary Tables S1–S5.

Based on the study hypotheses, a series of independent longitudinal path models was estimated. Specifically, separate models were constructed for each actigraphy-derived sleep index (TST, SE, and WASO) and each cognitive-control and ToM outcome. This modeling strategy was adopted to preserve statistical power and avoid model overparameterization relative to the available sample size.

The longitudinal path models were estimated using the full sample, as subgroup sizes were insufficient to support reliable diagnosis-specific or multigroup analyses. Consequently, the reported associations should be interpreted as reflecting overall longitudinal relationships across participants rather than diagnosis-specific mechanisms.

Overall, the bivariate correlation patterns indicated that TST showed the strongest and most consistent positive associations, particularly with ToM measures. SE also demonstrated significant positive correlations with several cognitive-control and ToM variables, although these were generally weaker than those observed for TST. WASO showed fewer and predominantly negative correlations across assessment waves. Among the ToM variables, the highest correlations with TST were observed for third-order ToM stories, non-literal speech 1, non-literal speech 2, Verbal Metaphors in Context, and TASIT—Part I (decoding of uncertain emotional expressions). Comparatively weaker, but significant correlations were also observed for TASIT Happy and TASIT Neutral performance.

The longitudinal path models provided a more stringent test of these associations by accounting for the temporal continuity (autoregressive effects) of both the sleep indices and the cognitive-control and ToM outcomes across assessment waves. Within this framework, prospective cross-lagged effects of the sleep indices were examined. Although all models showed an acceptable overall pattern of fit indices, the significant prospective effects differed substantially across sleep indices. TST demonstrated the broadest pattern of significant prospective associations, predicting subsequent performance across all ToM outcomes, with the exception of Nominal Metaphors in Context, whereas significant prospective effects for cognitive control were limited to the Tower Test Total Number of Problems and Verbal Fluency Test Condition IIIa. By contrast, SE demonstrated prospective associations with only two ToM outcomes (non-literal speech 1 and Verbal Metaphors in Context) and no cognitive-control outcomes, whereas WASO did not demonstrate significant prospective associations with either cognitive-control or ToM outcomes. Thus, although the overall longitudinal models were characterized by strong autoregressive continuity of both the sleep indices and the cognitive-control and ToM outcomes, only a subset of the prospective cross-lagged sleep–outcome paths remained statistically significant over time.

Given the large number of estimated models and the limited sample size relative to the number of study variables, estimating a single comprehensive longitudinal model including all sleep, cognitive control, and ToM variables was not considered appropriate. Such a model would have been highly parameterized, increasing the risk of unstable estimates and reduced statistical power. Therefore, a series of independent longitudinal path models was estimated. Because the TST models demonstrated the broadest and most consistent pattern of prospective associations, some of these models are presented in the main text, whereas the rest—TST, SE, and WASO models—are reported in Supplementary Material Tables S6–S9.

Across the estimated path models, the chi-squared tests were statistically significant, whereas the CFI and SRMR consistently indicated satisfactory model fit. Although the RMSEA exceeded conventional cut-offs, both the chi-squared statistic and the RMSEA are known to be sensitive in models with relatively few degrees of freedom and small samples. Therefore, model fit was evaluated based on the overall pattern of fit indices rather than on any single statistic [69,70].

**Figure 4:**
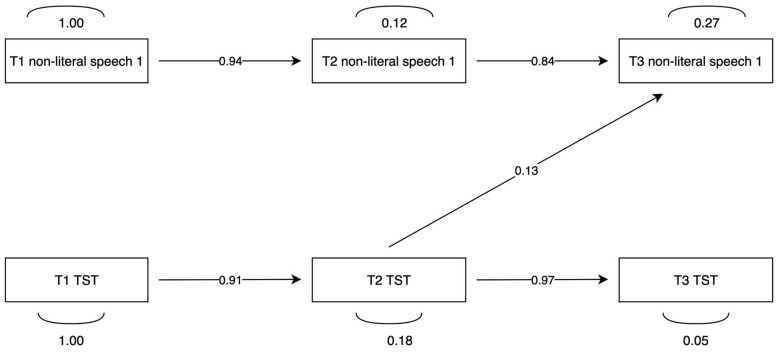
Longitudinal path model illustrating the associations between sleep duration (TST) and non-literal speech 1 (recognition of non-literal meaning) across the three assessment waves. Note. Standardized path coefficients (β) are shown.

Model A examined the longitudinal associations between TST and non-literal speech 1 across the three assessment waves. Although the chi-squared test was significant, χ^2^(7) = 25.70, *p* < 0.001, CFI = 0.99 and SRMR = 0.06 indicated satisfactory fit, RMSEA was 0.12 (95% CI [0.07, 0.18]). TST at T2 significantly predicted non-literal speech 1 performance at T3 (β = 0.128, *p* < 0.001) after controlling for prior task performance. The model explained 73.4% of the variance in T3 non-literal speech 1 performance. The corresponding path from T1 TST to T2 non-literal speech 1 was not significant (β = 0.036, *p* = 0.169). Figure 4 presents the simplified model, including statistically significant paths only. The complete model specifications, including non-significant paths, are provided in Supplementary Figures S1–S3.

Path Model B examined the longitudinal associations between TST and performance on the non-literal speech 2 task across the three assessment waves. Although the chi-squared test was significant, χ^2^(7) = 40.20, *p* < 0.001, the incremental fit indices suggested satisfactory model fit (CFI = 0.98, SRMR = 0.07), whereas the RMSEA was 0.16 (95% CI [0.12, 0.21]). TST at the second assessment wave significantly predicted non-literal speech 2 performance at T3 (standardized β = 0.134, *p* < 0.001) after controlling for prior levels of performance. Specifically, greater sleep duration at T2 was associated with better subsequent understanding of non-literal speech and speakers’ communicative intentions. The model explained 76.7% of the variance in T3 non-literal speech 2 performance. The corresponding path from T1 TST to T2 non-literal speech 2 was not statistically significant (β = −0.002, *p* = 0.927). Given the highly similar structure of the estimated path models, Figure 4 is presented as a representative example. The remaining path models are described in the text.

Model C examined the longitudinal associations between TST and performance on the Verbal Metaphors in Context task across the three assessment waves. Although the chi-squared test was significant, χ^2^(7) = 32.00, *p* < 0.001, the incremental fit indices suggested an overall satisfactory fit to the data (CFI = 0.98, SRMR = 0.03), whereas the RMSEA was 0.13 (90% CI [0.09, 0.18]). Results indicated that TST at the second assessment wave significantly predicted Verbal Metaphors in Context performance at T3 (standardized β = 0.177, *p* < 0.001), even after controlling for prior levels of metaphor comprehension performance. Specifically, greater sleep duration at T2 was associated with better subsequent ability to interpret metaphorical and abstract semantic content at T3. Conversely, reduced sleep duration predicted poorer subsequent performance over time, suggesting increasing difficulty in processing abstract and non-literal language. The model explained 59.1% of the variance in T3 Verbal Metaphors in Context performance. The corresponding path from T1 TST to T2 Verbal Metaphors in Context was not statistically significant (β = −0.043, *p* = 0.308).

Model C examined the longitudinal associations between TST and performance on the Verbal Metaphors in Context task across the three assessment waves. Although the chi-squared test was significant, χ^2^(7) = 31.0, *p* < 0.001, the incremental fit indices suggested satisfactory model fit (CFI = 0.98, SRMR = 0.03), whereas the RMSEA was 0.14 (95% CI [0.09, 0.19]). TST at the second assessment wave significantly predicted Verbal Metaphors in Context performance at T3 (standardized β = 0.178, *p* < 0.001), after controlling for prior levels of metaphor comprehension performance. Specifically, greater sleep duration at T2 was associated with better subsequent ability to interpret metaphorical and abstract semantic content. The model explained 58.7% of the variance in T3 Verbal Metaphors in Context performance. The corresponding path from T1 TST to T2 Verbal Metaphors in Context was not statistically significant (β = −0.043, *p* = 0.305).

Overall, the longitudinal path analyses demonstrated that TST showed the broadest and most consistent pattern of prospective associations across assessment waves. Specifically, TST significantly predicted subsequent performance on all ToM outcomes except Nominal Metaphors in Context and on two cognitive-control measures (Tower Test Total Number of Problems and Verbal Fluency Test Condition IIIa). By comparison, SE demonstrated prospective associations with only two ToM measures (non-literal speech 1 and Verbal Metaphors in Context), whereas WASO showed no significant prospective associations with either cognitive-control or ToM outcomes.

## 4. Discussion

### 4.1. Principal Findings

The present longitudinal study provides a comprehensive characterization of cognitive-control and ToM trajectories in individuals with aMCI and naMCI while simultaneously examining the prospective contribution of objectively measured sleep. The findings indicate that longitudinal cognitive change in MCI is not uniform but varies across cognitive domains. Although both cognitive-control and ToM performance declined over time, the most pronounced differences between the MCI subtypes emerged in higher-order executive and social–cognitive processes, with individuals with naMCI exhibiting the greatest impairment. The longitudinal path analyses further showed that the prospective associations between objectively measured sleep and later cognitive and ToM performance were not equivalent across sleep indices or cognitive and ToM domains. Among the examined actigraphy-derived sleep parameters, TST demonstrated the broadest and most consistent prospective associations, whereas SE showed only limited associations and WASO showed none. Notably, these prospective associations were considerably more extensive for ToM than for the cognitive-control measures examined in the present study. While previous research has largely focused on the relationship between sleep and global cognition, longitudinal evidence linking objectively measured sleep with social cognition in MCI remains scarce. This finding is of particular clinical relevance because ToM underpins successful interpersonal communication, interpretation of others’ intentions, and appropriate social behavior in everyday life. Consequently, preserving habitual sleep duration may have implications not only for cognitive health but also for maintaining social functioning and quality of life in older adults with MCI.

### 4.2. Interpretation of Cognitive-Control Trajectories

The present findings partially confirmed the first hypothesis by demonstrating progressive decline in cognitive-control performance across the three assessment waves in both MCI groups, whereas cognitively healthy older adults showed relatively stable trajectories. However, executive decline was not uniform in terms of its ability to distinguish the two MCI subtypes. Although healthy controls consistently outperformed both MCI groups across all executive measures, the clearest differentiation between aMCI and naMCI emerged in higher-order planning processes. Specifically, planning-related indices derived from the D-KEFS Tower Test, particularly the total number of problems and rule violations, distinguished the two clinical subtypes more consistently than measures of cognitive flexibility or inhibitory control, which primarily differentiated cognitively healthy individuals from participants with MCI.

This pattern is consistent with previous longitudinal evidence indicating that executive decline is a characteristic feature of MCI, whereas cognitively healthy aging is associated with relative stability over time [71]. The stable performance observed in healthy controls across planning, verbal fluency, and inhibitory control agrees with studies suggesting that core executive abilities remain relatively preserved during normative aging in the absence of significant neuropathology [72]. Accordingly, the trajectory observed in the healthy group provides an important benchmark for distinguishing pathological executive decline from normal age-related variability [73,74]. The present findings further extend this literature by suggesting that executive functions do not contribute equally to phenotypic differentiation within MCI. Rather than indicating a generalized dysexecutive syndrome, the results suggest that higher-order planning and performance-monitoring processes are particularly sensitive to the neurocognitive differences characterizing the two MCI subtypes [75].

Planning represents one of the most complex executive functions because it requires the dynamic integration of multiple cognitive processes, including working-memory updating, inhibitory control, cognitive flexibility, rule maintenance, and the sequential organization of goal-directed behavior [76]. Consequently, successful performance on the D-KEFS Tower Test depends on the coordinated operation of distributed executive-control networks rather than on a single cognitive ability [77,78]. Contemporary network models indicate that these higher-order executive processes are primarily supported by large-scale prefrontal–cingulate systems involving the dorsolateral prefrontal cortex, dorsal anterior cingulate cortex, and their reciprocal frontoparietal and frontosubcortical connections, which collectively support planning, performance monitoring, behavioral regulation, and error detection [79]. Within this neurocognitive framework, the finding that Tower Test performance—particularly the total number of problems and rule violations—provided the clearest distinction between aMCI and naMCI is compatible with the view that disruption of distributed executive-control networks represents an important feature of subtype-related executive vulnerability in MCI.

The selective sensitivity of planning measures suggests that higher-order executive functions may provide more informative markers of phenotypic variation within MCI than executive measures assessing cognitive flexibility or inhibitory control. Consistent with this interpretation, the two Tower Test indices that differentiated the MCI subtypes in the longitudinal analyses were also the only executive measures showing significant post hoc differences between aMCI and naMCI. This pattern is consistent with previous evidence indicating that naMCI is more frequently associated with disruption of frontosubcortical executive networks and vascular or mixed neuropathological processes, whereas aMCI is generally characterized by predominant medial temporal pathology during the earlier stages of disease progression [79,80,81]. Although the present study did not include neuroimaging or biomarker measures capable of directly testing these mechanisms, the observed longitudinal trajectories are compatible with current neurocognitive models proposing partly distinct neural substrates underlying the two MCI subtypes.

From a functional perspective, planning abilities support the organization of complex goal-directed behavior, including anticipating consequences, monitoring ongoing actions, maintaining task rules, and flexibly adapting behavior to changing environmental demands [82]. Consequently, impairment in these processes may contribute to reduced independence in everyday activities requiring problem solving, multitasking, and behavioral self-regulation. From a clinical perspective, the present findings therefore suggest that assessment of planning abilities may provide clinically meaningful information beyond global cognitive screening. Although brief cognitive screening instruments remain valuable for detecting cognitive impairment, they provide limited information regarding the qualitative profile of executive dysfunction. Incorporating measures of complex planning and performance monitoring may therefore improve the phenotypic characterization of MCI and complement traditional memory-oriented assessment. The longitudinal pattern observed in the present study further indicates that executive dysfunction in MCI is heterogeneous rather than uniform. Although both MCI groups showed progressive executive decline, planning and performance-monitoring measures provided the clearest behavioral distinction between aMCI and naMCI, highlighting their potential value as sensitive markers of subtype-related executive vulnerability. Whereas planning measures provided the clearest differentiation within executive functioning, the present findings suggest that even greater subtype-related differences emerge when higher-order social cognition is examined.

### 4.3. Interpretation of Theory of Mind Trajectories

The present findings supported the first hypothesis by demonstrating progressive decline in ToM performance across the three assessment waves in both MCI groups, whereas cognitively healthy older adults showed relatively stable trajectories. Compared with the executive-control findings discussed in the previous section, subtype-related differences emerged across a broader range of ToM measures, suggesting that higher-order social cognition may provide greater sensitivity for capturing phenotypic heterogeneity within MCI. Although healthy controls consistently achieved the highest performance, a clear performance gradient (HC > aMCI > naMCI) was observed for non-literal speech comprehension, verbal metaphor comprehension, proverb interpretation, and third-order mentalizing, indicating that these higher-order social–cognitive abilities are particularly sensitive to the neurocognitive differences characterizing the two MCI subtypes.

The relative stability observed in cognitively healthy older adults is consistent with evidence indicating that higher-order social–cognitive abilities remain comparatively preserved during normative aging, particularly when widespread neurodegenerative pathology is absent [83,84]. In contrast, the progressive decline observed across both MCI groups is consistent with previous evidence indicating that higher-order social cognition becomes increasingly vulnerable as neurodegenerative changes extend beyond medial temporal regions to involve distributed association networks supporting mental-state reasoning, contextual integration, and social inference [25,85]. Rather than reflecting normal age-related change, the longitudinal trajectories identified in the present study are therefore compatible with progressive disruption of higher-order social–cognitive processes accompanying MCI.

The distinction between the two MCI subtypes became most evident in tasks requiring the interpretation of non-literal language and communicative intentions. In particular, non-literal speech 2 provided the clearest behavioral differentiation between aMCI and naMCI. Unlike non-literal speech 1, which primarily requires recognizing that an utterance is inappropriate or non-literal, non-literal speech 2 additionally requires reconstructing the speaker’s intended meaning through the integration of contextual information, pragmatic language processing, and mental-state reasoning [86]. The greater impairment observed in individuals with naMCI therefore appears to reflect difficulties extending beyond language comprehension itself, involving the coordinated interpretation of contextual, semantic, and interpersonal information. This interpretation is consistent with evidence suggesting that higher-order pragmatic language and communicative inference are particularly vulnerable to the broader neurocognitive changes accompanying non-amnestic MCI [87,88].

The broader pattern observed across the ToM measures further suggests that successful social inference depends on the coordinated interaction of multiple cognitive systems rather than on a single specialized social–cognitive process. Contemporary neurocognitive models conceptualize ToM as an emergent function supported by the interaction of the mentalizing network—including the medial prefrontal cortex, temporoparietal junction, posterior superior temporal sulcus, temporal poles, and precuneus—with semantic-language systems and executive-control networks responsible for contextual updating, cognitive flexibility, and behavioral regulation [89,90]. Although the present study did not include neuroimaging measures capable of directly examining these mechanisms, this framework provides a plausible explanation for why higher-order ToM differentiated the MCI subtypes more consistently than executive-control measures. Tasks requiring the simultaneous integration of contextual, semantic, and mental-state information may simply be more sensitive to the distributed network dysfunction characterizing MCI than tasks relying on more circumscribed cognitive operations.

The profile observed in individuals with aMCI remained consistently intermediate between healthy controls and the naMCI group throughout the follow-up period. Because the present study did not directly assess memory functioning or underlying neuropathology, the mechanisms responsible for this intermediate profile cannot be determined conclusively. Nevertheless, previous evidence suggests that the predominantly medial temporal pathology associated with aMCI may gradually extend beyond memory systems to influence broader associative networks supporting complex social cognition [91]. This interpretation is compatible with the present findings, in which ToM performance declined over time in both MCI groups, but remained consistently more impaired in naMCI.

The longitudinal pattern was not identical across all ToM measures. Metaphor and proverb comprehension demonstrated progressively greater differentiation between the diagnostic groups across the three assessments, whereas third-order ToM stories showed significant group differences without a significant group × time interaction. Similarly, TASIT emotion-recognition measures demonstrated significant longitudinal change, but comparatively smaller interaction effects. This variability suggests that different components of social cognition differ in their sensitivity to longitudinal progression. Within the present sample, tasks placing greater demands on pragmatic language processing, contextual integration, and communicative inference appeared to be particularly sensitive to subtype-related differences. At the same time, these tasks also place demands on semantic knowledge and broader cognitive abilities; therefore, their greater longitudinal sensitivity is likely to reflect the combined contribution of social inference, pragmatic language processing, and semantic integration rather than ToM processes in isolation [92,93].

Successful everyday communication depends not only on remembering information but also on accurately interpreting communicative intentions, understanding figurative language, and integrating contextual social cues. The greater impairment observed in these higher-order ToM abilities, particularly in individuals with naMCI, suggests that social–cognitive decline may contribute to everyday communication difficulties that are not readily captured by conventional cognitive assessment. Problems understanding indirect speech, appreciating implied meaning, or adapting responses to changing conversational contexts may reduce communicative effectiveness and social participation despite relatively preserved basic language abilities [94]. These observations support the inclusion of comprehensive ToM assessment alongside traditional neuropsychological measures to provide a more complete characterization of the functional consequences of cognitive decline in MCI.

### 4.4. Sleep–Theory of Mind Associations

The longitudinal path analyses partially supported the second hypothesis by demonstrating that objective sleep parameters differed substantially in their prospective associations with subsequent cognitive functioning. Although the bivariate analyses revealed significant associations between all three actigraphy-derived sleep indices and several cognitive-control and ToM measures, the longitudinal path models provided a considerably more stringent test by accounting for the strong temporal stability (autoregressive continuity) of both the sleep indices and the cognitive outcomes across assessment waves. Within this framework, TST emerged as the objective sleep parameter showing the broadest and most consistent pattern of prospective associations, whereas SE retained only a limited number of significant longitudinal associations and WASO showed none. Specifically, TST prospectively predicted subsequent performance across a broad range of ToM measures, including non-literal speech comprehension, verbal metaphor comprehension, proverb interpretation, third-order mentalizing, and TASIT emotion-recognition measures, whereas prospective associations with cognitive control were limited to only two executive measures (Tower Test total number of problems and verbal fluency test, Condition IIIa). SE retained prospective associations with only two ToM measures (non-literal speech 1 and Verbal Metaphors in Context), while WASO did not predict subsequent performance on either cognitive-control or ToM outcomes. Importantly, these associations remained significant despite the strong temporal stability characterizing both sleep and cognitive performance across the three assessment waves, suggesting that habitual sleep duration explained unique variance in subsequent social–cognitive functioning beyond the stability of the examined constructs.

This pattern is noteworthy because previous research has primarily focused on the relationship between sleep and global cognitive functioning or memory, whereas considerably less attention has been devoted to social cognition. The present findings extend this literature by demonstrating that after accounting for temporal continuity, objectively measured sleep duration remained prospectively associated predominantly with higher-order ToM abilities rather than with the executive-control functions assessed in the present study. Importantly, these findings do not indicate that executive functioning is unrelated to sleep. Rather, they suggest that the prospective associations with the executive measures included in the present study were comparatively limited, whereas the most robust longitudinal relationships involved complex social–cognitive abilities. Furthermore, the absence of comparable prospective associations for SE and WASO suggests that objective sleep parameters may differ in their longitudinal relevance for cognitive functioning. Whereas SE has often been linked more closely to subjective alertness and daytime fatigue [95] and WASO primarily reflects sleep fragmentation associated with attentional and memory processes [96], habitual sleep duration may be more closely associated with the maintenance of demanding higher-order social–cognitive abilities [97,98]. Nevertheless, the observed prospective effects of TST were modest in magnitude (standardized β ≈ 0.13–0.18) and should therefore be interpreted as indicating small, but reliable longitudinal associations rather than strong predictive effects.

The specific ToM measures that remained prospectively associated with TST provide further insight into the nature of this relationship. The strongest longitudinal associations involved tasks requiring comprehension of non-literal language, interpretation of communicative intentions, metaphorical and proverbial meaning, third-order mental-state reasoning, and the decoding of subtle emotional expressions. These abilities extend beyond basic emotion recognition and rely on the integration of contextual information, semantic knowledge, pragmatic language processing, executive regulation, and flexible mental-state attribution [87,99]. Rather than reflecting isolated social–cognitive processes, these tasks represent complex integrative functions requiring the coordinated operation of multiple cognitive systems, including executive control, semantic processing, pragmatic language, and mental-state reasoning [100,101]. This interpretation is consistent with contemporary neurocognitive models conceptualizing TST as an emergent function supported by the interaction of the mentalizing network, including the medial prefrontal cortex, temporoparietal junction, posterior superior temporal sulcus, temporal poles, and precuneus, with semantic-language systems and executive-control networks responsible for contextual updating, cognitive flexibility, and behavioral regulation [100,102]. Although these neural mechanisms were not directly examined in the present study, they provide a plausible framework for interpreting why objectively measured sleep duration was more consistently associated with complex social inference than with the executive-control measures examined here.

Growing evidence indicates that insufficient sleep is associated with alterations in neural systems supporting emotional regulation, salience detection, executive control, and social information processing [97,103,104,105]. Experimental and neuroimaging studies further suggest that sleep loss alters functional connectivity within the default mode, salience, and frontoparietal control networks, which collectively support the integration of internally generated mental-state representations with external contextual and socially relevant information [97,104,105]. Because successful social inference depends on the coordinated operation of these interacting systems, habitual reductions in sleep duration may reduce the efficiency with which contextual cues, communicative intentions, and mental-state representations are integrated during demanding interpersonal situations. Contemporary models of mentalization similarly conceptualize social cognition as a higher-order integrative process emerging from the interaction of cognitive and affective systems rather than from executive functioning alone [106]. Within this framework, sleep-related alterations in emotional regulation, affective responsiveness, and the processing of socially salient information may contribute to changes in higher-order social cognition through pathways that extend beyond the executive processes assessed in the present study [107,108]. Although the present study did not include polysomnographic or neuroimaging measures, these mechanisms may plausibly influence the ability to decode subtle emotional expressions, infer communicative intentions, and integrate socially relevant contextual information—abilities that were prominently represented among the ToM measures showing prospective associations with habitual sleep duration. This interpretation is consistent with previous evidence indicating that insufficient sleep adversely affects emotional processing and interpersonal functioning, both of which are fundamental components of successful higher-order social [109,110].

Recent evidence further indicates that sleep loss may impair social functioning through alterations in affective and interpersonal processing, potentially involving frontolimbic and distributed social-brain networks supporting emotional regulation and complex social inference [1]. Additional mechanisms including alterations in arousal regulation, physiological stress responses, and other biological processes accompanying MCI may also contribute to the observed longitudinal outcomes [111]. Although the present findings are compatible with these proposed mechanisms, the behavioral data alone cannot determine the biological pathways underlying the observed longitudinal associations. Consequently, the present results should be interpreted as demonstrating prospective behavioral relationships rather than identifying the neurobiological mechanisms through which habitual sleep duration may influence higher-order social cognition.

From a clinical perspective, these findings may be particularly relevant because the social–cognitive abilities that were prospectively associated with sleep duration underpin successful everyday communication rather than performance on neuropsychological tests alone. Understanding indirect speech, inferring communicative intentions, interpreting metaphorical language, and accurately decoding subtle emotional expressions are fundamental for maintaining effective interpersonal relationships, adapting behavior to changing social contexts, and participating successfully in everyday interactions. Difficulties in these abilities may contribute to interpersonal misunderstandings, reduced social participation, diminished communicative confidence, and ultimately poorer quality of life in older adults with MCI [88,101]. Thus, the present findings broaden current perspectives linking sleep primarily to memory and executive functioning by suggesting that habitual sleep duration may also represent a clinically relevant longitudinal marker of vulnerability for higher-order social–cognitive abilities that directly support everyday interpersonal functioning [112].

The longitudinal pattern observed in the present study suggests that the relationship between objectively measured sleep and cognition is not uniformly distributed across cognitive domains. After accounting for the substantial temporal stability of both sleep and cognitive performance, habitual sleep duration showed its most consistent prospective associations with ToM, whereas the executive-control measures examined in the present study demonstrated comparatively fewer longitudinal relationships. Although these findings should not be interpreted as evidence of causality, they suggest that the longitudinal consequences of insufficient sleep may extend beyond traditional cognitive domains to encompass the complex social–cognitive abilities that support successful communication, interpersonal understanding, and everyday social functioning during the early stages of cognitive decline. By highlighting higher-order social cognition as a domain that appears particularly sensitive to habitual sleep duration, the present findings broaden current perspectives linking sleep primarily to memory and executive functioning and underscore the importance of considering social cognition in future longitudinal research on aging and MCI. Future studies integrating objective sleep assessment with polysomnography, multimodal neuroimaging, and larger well-characterized clinical cohorts will be important for clarifying the neurobiological mechanisms underlying these associations and determining whether preserving habitual sleep duration contributes to maintaining social–cognitive functioning in individuals at increased risk for dementia.

## 5. Conclusions

The present study demonstrated distinct longitudinal trajectories of cognitive control and ToM across healthy aging, aMCI, and naMCI. Whereas cognitively healthy older adults showed relatively stable performance across executive and social–cognitive domains, both MCI groups exhibited progressive decline over time, with individuals with naMCI showing the greatest impairment, particularly in planning, rule monitoring, and complex ToM abilities involving non-literal language, communicative intentions, and abstract social inference. These findings support the view that aMCI and naMCI represent partly distinct neurocognitive phenotypes characterized by different patterns of executive and social–cognitive vulnerability.

The study further demonstrated that objectively measured habitual sleep duration was prospectively associated predominantly with subsequent higher-order ToM performance, whereas longitudinal associations with the executive-control measures examined in the present study were comparatively limited. By extending the investigation of sleep beyond global cognition and memory to higher-order social cognition, the present findings suggest that habitual sleep duration may represent a clinically relevant longitudinal marker of vulnerability for complex interpersonal abilities that support everyday communication and social functioning in older adults with MCI. Although these longitudinal associations should not be interpreted as evidence of causality, they highlight the importance of considering social cognition alongside executive functioning when investigating the cognitive consequences of insufficient sleep in populations at increased risk for dementia.

## 6. Limitations

Although the present study employed a longitudinal design and incorporated objective sleep assessment using actigraphy, several methodological considerations should be acknowledged when interpreting the findings. First, although actigraphy provides an ecologically valid and non-invasive method for monitoring habitual sleep–wake patterns under naturalistic conditions, it does not capture detailed sleep architecture, including rapid eye movement (REM) sleep and slow-wave sleep, which may be particularly relevant to neurodegenerative processes. Participants were screened for sleep disorders using the STOP-Bang Questionnaire, and individuals with diagnosed sleep disorders or sleep-related medication use were excluded. Nevertheless, undiagnosed sleep disorders, particularly obstructive sleep apnea, cannot be entirely ruled out because polysomnography was not performed. Second, the absence of structural neuroimaging data and biological biomarkers (e.g., amyloid-β or tau biomarkers, or positron emission tomography [PET]) limited the ability to relate the observed behavioral findings directly to the underlying neurobiological mechanisms associated with MCI. Third, the relatively small subgroups precluded reliable estimation of longitudinal path models separately for the aMCI and naMCI groups. Consequently, the structural equation models were estimated using the full sample and should therefore be interpreted as reflecting longitudinal associations across the overall sample rather than subtype-specific mechanisms. Future studies including larger diagnostic subgroups will be needed to determine whether the observed sleep–ToM associations differ across MCI subtypes. Fourth, the present analyses focused on average longitudinal trajectories at the group level and did not estimate individual trajectories of change. Future studies with larger samples and additional follow-up assessments should examine inter-individual variability in cognitive and social–cognitive decline. The fifth limitation concerns the interpretation of several higher-order ToM measures. Tasks involving non-literal language, metaphor comprehension, and proverb interpretation also place substantial demands on language abilities and broader cognitive resources. Accordingly, the observed longitudinal associations may not exclusively reflect ToM processes, but also the broader linguistic, semantic, and executive demands required for successful task performance. Future studies incorporating comprehensive language assessment would help to further disentangle these contributions. Sixth, psychometric evaluation of the complete Natsopoulos’ ToM battery is ongoing. Although psychometric evidence is already available for several individual components of the battery [60,61], further validation of the complete instrument will strengthen its application in future longitudinal studies. Also, participants with MCI were recruited from specialized memory services and had received their diagnosis within the previous two years. Consequently, the findings primarily reflect relatively early and well-characterized stages of MCI and may not fully generalize to individuals with more advanced disease or those not receiving specialized clinical follow-up. Finally, because the present study was observational, the reported longitudinal associations should not be interpreted as evidence of causality. Although the temporal ordering afforded by the longitudinal design strengthens inference regarding prospective relationships, experimental and intervention studies will be required to determine whether improving habitual sleep contributes directly to preserving higher-order social cognition in individuals with MCI. Similarly, the absence of detailed measures of fatigue, or daytime sleepiness precludes evaluation of whether these factors may have contributed to the observed associations between sleep duration and higher-order social cognition.

## 7. Future Implications

The present findings open several important avenues for future research. Longitudinal studies integrating objective sleep assessment with structural and functional neuroimaging, polysomnographic measures of sleep architecture, and molecular biomarkers of neurodegeneration will be essential for clarifying the neurobiological mechanisms linking habitual sleep duration with higher-order social cognition across the MCI continuum. Such multimodal approaches may determine whether the observed longitudinal associations reflect alterations in distributed neural systems supporting social cognition or broader neurodegenerative processes that evolve alongside sleep disturbances. Future research should also determine whether these longitudinal associations differ across MCI phenotypes. Larger longitudinal cohorts will allow adequately powered subgroup analyses and multigroup structural equation modeling to establish whether amnestic and non-amnestic MCI follow distinct sleep–cognition trajectories. Examining the potential moderating role of demographic and clinical characteristics, including sex, cognitive reserve, vascular burden, and other comorbidities, may further improve understanding of the factors contributing to individual variability in cognitive and social–cognitive decline. Finally, intervention studies targeting habitual sleep duration and overall sleep health are needed to determine whether improving sleep is associated with better preservation of higher-order social cognition over time. Establishing whether sleep represents a modifiable risk factor, rather than solely a longitudinal marker of social–cognitive vulnerability, would have important implications for early intervention strategies aimed at preserving everyday communication, interpersonal functioning, and quality of life in older adults at increased risk of dementia.

## Figures and Tables

**Table 1:** Participants’ demographic characteristics.

	HC	aMCI	naMCI
	Mean (SD)	Mean (SD)	Mean (SD)
Age	70.28 (5.02), 60–83	71.09 (4.98), 65–85	69.07 (4.18), 63–79
Education	11.98 (3.09), 6–18	12.41 (3.18), 6–18	12.57 (3.39), 6–23
Gender (f/m)	36/10	51/24	39/19

**Table 2:** Mixed-design ANOVA results for D-KEFS Tower Test, Verbal Fluency Test, and Color–Word Interference Test variables.

Variable	Effect	F (df)	*p*	η^2^
TT: Total achievement score	Time	39.83 (1.32, 231.74)	<0.001	0.18
	Group	66.52 (2, 176)	<0.001	0.43
	Time × Group	17.59 (2.63, 231.74)	<0.001	0.16
TT: Total number of problems	Time	55.38 (1.70, 298.55)	<0.001	0.23
	Group	57.77 (2, 176)	<0.001	0.39
	Time × Group	20.44 (3.39, 298.55)	<0.001	0.18
TT: Total number of rule violations	Time	126.56 (1.73, 303.72)	<0.001	0.41
	Group	131.08 (2, 176)	<0.001	0.59
	Time × Group	29.51 (3.45, 303.72)	<0.001	0.25
VFT: Condition (IIIa)	Time	187.85 (1.81, 318.41)	<0.001	0.52
	Group	15.20 (2, 176)	<0.001	0.15
	Time × Group	51.18 (3.62, 318.41)	<0.001	0.37
VFT: Condition (IIIb)	Time	184.28 (1.80, 315.95)	<0.001	0.51
	Group	9.47 (2, 176)	<0.001	0.10
	Time × Group	55.62 (3.59, 315.95)	<0.001	0.39
CWIT: Condition (III)	Time	83.20 (1.81, 318.97)	<0.001	0.32
	Group	4.64 (2, 176)	0.011	0.05
	Time × Group	14.77 (3.63, 318.97)	<0.001	0.14
CWIT: Condition (IV)	Time	112.42 (1.72, 302.51)	<0.001	0.39
	Group	1.70 (2, 176)	0.186	0.02
	Time × Group	11.13 (3.44, 302.51)	<0.001	0.11

Note: 1. TT: Tower Test; VFT: Verbal Fluency Test; CWIT: Color–Word Interference Test from the Delis–Kaplan Executive Function System (D-KEFS). 2. Bonferroni-adjusted significance thresholds were *p* < 0.007 for cognitive-control outcomes.

**Table 3:** Means and standard deviations for cognitive-control variables by group and time point.

Variable	Time	HCs (n = 46) M (SD)	aMCI (n = 75) M (SD)	naMCI (n = 58) M (SD)
TT: Total achievement score	T1	17.87 (2.21)	13.45 (2.84)	13.43 (2.71)
	T2	18.13 (2.22)	13.28 (2.73)	12.50 (2.60)
	T3	17.89 (2.10)	12.89 (2.67)	12.16 (2.71)
TT: Total number of problems	T1	8.80 (0.69)	7.40 (1.38)	6.74 (1.38)
	T2	8.80 (0.69)	7.32 (1.39)	6.34 (1.22)
	T3	8.80 (0.69)	7.11 (1.33)	6.00 (1.08)
TT: Total number of rule violations	T1	0.46 (0.69)	3.23 (3.31)	9.12 (3.77)
	T2	0.33 (0.52)	3.44 (3.26)	9.57 (3.87)
	T3	0.50 (0.66)	4.16 (3.23)	10.79 (4.11)
VFT: Condition (IIIa)	T1	15.30 (2.61)	14.08 (3.06)	14.62 (3.05)
	T2	16.43 (2.53)	13.44 (2.66)	13.21 (2.80)
	T3	15.39 (2.63)	12.05 (2.61)	11.66 (2.81)
VFT: Condition (IIIb)	T1	22.74 (3.89)	20.88 (4.46)	21.95 (4.65)
	T2	23.98 (3.75)	20.16 (4.05)	20.59 (4.39)
	T3	22.93 (3.75)	18.81 (3.84)	19.03 (4.51)
CWIT: Condition (III)	T1	0.87 (1.38)	1.19 (1.53)	0.93 (1.11)
	T2	0.87 (1.26)	1.47 (1.53)	1.59 (1.31)
	T3	1.00 (1.25)	2.21 (1.81)	2.38 (1.61)
CWIT: Condition (IV)	T1	2.04 (1.46)	2.44 (2.93)	2.09 (1.93)
	T2	2.04 (1.30)	2.72 (2.85)	2.78 (1.71)
	T3	2.43 (1.29)	3.57 (3.16)	3.67 (2.03)

Note: TT: Tower Test; VFT: Verbal Fluency Test; CWIT: Color–Word Interference Test from the Delis–Kaplan Executive- Function System (D-KEFS).

**Table 4:** Post hoc comparisons for cognitive-control variables across groups and time.

Variable	Comparison Type	Comparison	Mean Diff (I–J)	*p*
TT: Total achievement score	Group	HC vs. aMCI	4.76	<0.001
		HC vs. naMCI	5.27	<0.001
		aMCI vs. naMCI	0.51	0.746
	Time	T1 vs. T2	0.28	<0.001
		T1 vs. T3	0.61	<0.001
		T2 vs. T3	0.32	<0.001
TT: Total number of problems	Group	HC vs. aMCI	1.53	<0.001
		HC vs. naMCI	2.44	<0.001
		aMCI vs. naMCI	0.91	<0.001
	Time	T1 vs. T2	0.16	<0.001
		T1 vs. T3	0.35	<0.001
		T2 vs. T3	0.19	<0.001
TT: Total number of rule violations	Group	HC vs. aMCI	−3.18	<0.001
		HC vs. naMCI	−9.40	<0.001
		aMCI vs. naMCI	−6.22	<0.001
	Time	T1 vs. T2	−0.18	0.001
		T1 vs. T3	−0.88	<0.001
		T2 vs. T3	−0.71	<0.001
VFT: Condition (IIIa)	Group	HC vs. aMCI	2.52	<0.001
		HC vs. naMCI	2.55	<0.001
		aMCI vs. naMCI	0.03	1.000
	Time	T1 vs. T2	0.31	<0.001
		T1 vs. T3	1.64	<0.001
		T2 vs. T3	1.33	<0.001
VFT: Condition (IIIb)	Group	HC vs. aMCI	3.27	<0.001
		HC vs. naMCI	2.69	0.003
		aMCI vs. naMCI	−0.57	1.000
	Time	T1 vs. T2	0.28	0.002
		T1 vs. T3	1.60	<0.001
		T2 vs. T3	1.31	<0.001
CWIT: Condition (III)	Group	HC vs. aMCI	−0.71	0.019
		HC vs. naMCI	−0.72	0.026
		aMCI vs. naMCI	−0.01	1.000
	Time	T1 vs. T2	−0.31	<0.001
		T1 vs. T3	−0.87	<0.001
		T2 vs. T3	−0.56	<0.001
CWIT: Condition (IV)	Group	HC vs. aMCI	−0.74	0.248
		HC vs. naMCI	−0.67	0.401
		aMCI vs. naMCI	0.07	1.000
	Time	T1 vs. T2	−0.32	<0.001
		T1 vs. T3	−1.04	<0.001
		T2 vs. T3	−0.71	<0.001

Note: TT: Tower Test; VFT: Verbal Fluency Test; CWIT: Color–Word Interference Test from the Delis–Kaplan Executive Function System (D-KEFS).

**Table 5:** Mixed-design ANOVA results for theory of mind measures across groups and time.

Variable	Effect	F (df)	*p*	η^2^
Non-literal speech 1	Time	81.91 (1.39, 244.48)	<0.001	0.31
	Group	82.33 (2, 176)	<0.001	0.48
	Time × Group	23.43 (2.78, 244.48)	<0.001	0.21
Non-literal speech 2	Time	133.92 (1.27, 223.81)	<0.001	0.43
	Group	167.18 (2, 176)	<0.001	0.65
	Time × Group	5.77 (2.54, 223.81)	0.002	0.06
Verbal Metaphors in Context	Time	194.76 (1.93, 339.57)	<0.001	0.52
	Group	18.33 (2, 176)	<0.001	0.17
	Time × Group	21.47 (3.86, 339.57)	<0.001	0.19
Nominal Metaphors in Context	Time	111.42 (1.71, 301.48)	<0.001	0.39
	Group	33.20 (2, 176)	<0.001	0.27
	Time × Group	9.82 (3.43, 301.48)	<0.001	0.10
Proverbs in Context	Time	208.95 (1.60, 281.42)	<0.001	0.54
	Group	52.62 (2, 176)	<0.001	0.37
	Time × Group	16.46 (3.20, 281.42)	<0.001	0.16
Third-order ToM stories	Time	71.45 (1.48, 260.94)	<0.001	0.29
	Group	96.15 (2, 176)	<0.001	0.52
	Time × Group	1.98 (2.97, 260.94)	0.118	0.02
TASIT-PART 1 (Neutral)	Time	30.72 (1.68, 295.58)	<0.001	0.15
	Group	18.31 (2, 176)	<0.001	0.17
	Time × Group	4.06 (3.36, 295.58)	0.006	0.04
TASIT—Part 1 (Happy)	Time	66.27 (1.63, 287.21)	<0.001	0.27
	Group	18.97 (2, 176)	<0.001	0.18
	Time × Group	9.17 (3.26, 287.21)	<0.001	0.09
TASIT—Part 1 (Decoding of uncertain emotional expressions)	Time	49.22 (1.47, 258.58)	<0.001	0.22
	Group	34.00 (2, 176)	<0.001	0.28
	Time × Group	6.24 (2.94, 258.58)	<0.001	0.07

Note. Bonferroni-adjusted significance threshold for ToM outcomes was *p* < 0.006.

**Table 6:** Means and standard deviations for ToM variables by group and time point.

Variable	Time	HCs (n = 46) M (SD)	aMCI (n = 75) M (SD)	naMCI (n = 58) M (SD)
Non-literal speech 1	T1	4.00 (0.00)	3.51 (0.69)	2.66 (1.00)
	T2	3.98 (0.15)	3.41 (0.76)	2.47 (0.94)
	T3	3.98 (0.15)	3.03 (0.92)	1.69 (0.98)
Non-literal speech 2	T1	10.00 (1.48)	7.52 (2.29)	3.55 (2.02)
	T2	9.50 (1.74)	7.19 (2.26)	3.29 (2.01)
	T3	8.85 (1.83)	5.36 (2.23)	1.79 (1.79)
Verbal Metaphors in Context	T1	3.67 (0.47)	3.47 (0.72)	3.24 (0.82)
	T2	3.48 (0.51)	3.31 (0.72)	2.93 (0.70)
	T3	3.41 (0.50)	2.73 (0.58)	2.24 (0.68)
Nominal Metaphors in Context	T1	3.74 (0.49)	2.96 (0.86)	2.76 (0.94)
	T2	3.59 (0.54)	2.80 (0.81)	2.64 (0.87)
	T3	3.48 (0.55)	2.45 (0.78)	2.03 (0.77)
Proverbs in Context	T1	7.35 (0.74)	6.31 (1.39)	5.43 (1.31)
	T2	7.20 (0.78)	6.05 (1.28)	5.07 (1.17)
	T3	7.00 (0.70)	5.31 (1.25)	4.33 (1.08)
Third-order ToM stories	T1	2.83 (0.38)	1.80 (0.89)	1.12 (0.68)
	T2	2.72 (0.46)	1.68 (0.77)	1.12 (0.68)
	T3	2.52 (0.51)	1.44 (0.66)	0.72 (0.59)
ΤASIT—Part 1 (Neutral)	T1	0.91 (0.29)	0.77 (0.45)	0.59 (0.50)
	T2	0.91 (0.29)	0.65 (0.48)	0.45 (0.50)
	T3	0.85 (0.36)	0.41 (0.50)	0.29 (0.46)
ΤASIT—Part 1 (Happy)	T1	1.91 (0.29)	1.76 (0.49)	1.76 (0.43)
	T2	1.96 (0.21)	1.67 (0.55)	1.40 (0.62)
	T3	1.78 (0.42)	1.21 (0.64)	0.98 (0.76)
ΤASIT—Part 1 (Decoding of uncertain emotional expressions)	T1	5.54 (0.59)	4.91 (1.04)	4.34 (0.81)
	T2	5.54 (0.59)	4.81 (1.02)	4.29 (0.77)
	T3	5.43 (0.62)	4.60 (1.03)	3.86 (0.78)

**Table 7:** Post hoc comparisons for ToM variables across groups and time.

Variable	Comparison Type	Comparison	Mean Diff (I–J)	*p*
Non-literal speech 1	Group	HC vs. aMCI	0.67	<0.001
		HC vs. naMCI	1.72	<0.001
		aMCI vs. naMCI	1.05	<0.001
	Time	T1 vs. T2	0.10	<0.001
		T1 vs. T3	0.49	<0.001
		T2 vs. T3	0.39	<0.001
Non-literal speech 2	Group	HC vs. aMCI	2.76	<0.001
		HC vs. naMCI	6.57	<0.001
		aMCI vs. naMCI	3.81	<0.001
	Time	T1 vs. T2	0.36	<0.001
		T1 vs. T3	1.69	<0.001
		T2 vs. T3	1.33	<0.001
Verbal Metaphors in Context	Group	HC vs. aMCI	0.35	0.006
		HC vs. naMCI	0.72	<0.001
		aMCI vs. naMCI	0.36	0.002
	Time	T1 vs. T2	0.22	<0.001
		T1 vs. T3	0.67	<0.001
		T2 vs. T3	0.44	<0.001
Nominal Metaphors in Context	Group	HC vs. aMCI	0.86	<0.001
		HC vs. naMCI	1.12	<0.001
		aMCI vs. naMCI	0.26	0.123
	Time	T1 vs. T2	0.14	<0.001
		T1 vs. T3	0.50	<0.001
		T2 vs. T3	0.35	<0.001
Proverbs in Context	Group	HC vs. aMCI	1.29	<0.001
		HC vs. naMCI	2.24	<0.001
		aMCI vs. naMCI	0.95	<0.001
	Time	T1 vs. T2	0.26	<0.001
		T1 vs. T3	0.82	<0.001
		T2 vs. T3	0.56	<0.001
Third-order ToM stories	Group	HC vs. aMCI	1.05	<0.001
		HC vs. naMCI	1.70	<0.001
		aMCI vs. naMCI	0.65	<0.001
	Time	T1 vs. T2	0.08	<0.001
		T1 vs. T3	0.35	<0.001
		T2 vs. T3	0.28	<0.001
TASIT—Part 1 (Neutral)	Group	HC vs. aMCI	0.28	<0.001
		HC vs. naMCI	0.45	<0.001
		aMCI vs. naMCI	0.17	0.031
	Time	T1 vs. T2	0.09	<0.001
		T1 vs. T3	0.24	<0.001
		T2 vs. T3	0.15	<0.001
TASIT—Part 1 (Happy)	Group	HC vs. aMCI	0.34	<0.001
		HC vs. naMCI	0.51	<0.001
		aMCI vs. naMCI	0.17	0.071
	Time	T1 vs. T2	0.14	<0.001
		T1 vs. T3	0.48	<0.001
		T2 vs. T3	0.35	<0.001
TASIT—Part 1 (Decoding of uncertain emotional expressions)	Group	HC vs. aMCI	0.73	<0.001
		HC vs. naMCI	1.34	<0.001
		aMCI vs. naMCI	0.61	<0.001
	Time	T1 vs. T2	0.05	0.065
		T1 vs. T3	0.30	<0.001
		T2 vs. T3	0.25	<0.001

**Table 8:** Means and standard deviations of actigraphy-derived sleep indices across the three assessment waves by diagnostic group.

Sleep Parameter	Assessment Wave	Means (SD) HC (n = 46)	Means (SD) aMCI (n = 75)	Means (SD) naMCI (n = 58)
TST	Baseline Evaluation (T1)	370.48 (60.41)	343.68 (66.41)	326.91 (62.25)
	2nd Evaluation (T2)	407.83 (59.33)	343.45 (65.50)	324.69 (59.54)
	3rd Evaluation (T3)	395.91 (64.41)	336.00 (64.43)	313.52 (56.16)
SE	Baseline Evaluation (T1)	77.61 (8.58)	77.76 (10.58)	76.74 (8.91)
	2nd Evaluation (T2)	80.89 (7.60)	77.48 (10.30)	75.90 (8.42)
	3rd Evaluation (T3)	79.89 (7.71)	75.77 (10.08)	73.74 (8.34)
WASO	Baseline Evaluation (T1)	79.00 (43.58)	73.48 (50.03)	80.24 (46.36)
	2nd Evaluation (T2)	73.11 (41.11)	74.69 (48.63)	83.10 (45.27)
	3rd Evaluation (T3)	77.54 (41.82)	79.84 (49.31)	89.07 (46.38)

## Data Availability

The data presented in this study are available on request from the corresponding author, as they are part of my doctoral dissertation, which has not yet been fully published.

## Supplementary Materials

The following supporting information can be downloaded at https://www.mdpi.com/article/10.3390/jcm15155780/s1; Supplementary Tables S1–S9: Complete correlation matrices among sleep, cognitive-control, and theory of mind variables across assessment waves, as well as model fit indices and standardized prospective path coefficients for the longitudinal path models. Supplementary Figures S1–S3: Longitudinal path models illustrating the associations between total sleep time (TST) and non-literal speech 1, non-literal speech 2, and verbal metaphors in context across the three assessment waves.

## References

[1] Hyndych A., El-Abassi R., Mader E.C. (2025). The role of sleep and the effects of sleep loss on cognitive, affective, and behavioral processes. Cureus.

[2] Fernandez F.X., Grandner M.A. (2025). A comprehensive model for the converging biologies that underpin the homeostatic sleep signal. Sleep Med..

[3] Koutsimani P., Kosmidis M. (2026). Sleep disturbances and cognitive decline in older adults: A narrative review. Hell. J. Psychol..

[4] Bergamo G., Liguori C. (2025). Are sleep disturbances modifiable risk factors for mild cognitive impairment and dementia? A systematic review of large studies. Sleep Breath..

[5] Alster P., Madetko-Alster N., Migda A., Migda B., Kutyłowski M., Królicki L., Friedman A. (2023). Sleep disturbances in progressive supranuclear palsy syndrome (PSPS) and corticobasal syndrome (CBS). Neurol. Neurochir. Pol..

[6] Martiadis V., Pessina E., Martini A., Marzolla M., Bergesio C., Barbaro F., Cavallo A., Raffone F., Cattaneo C.I. (2026). Insomnia severity in psychiatric outpatients: Real-world Insomnia Severity Index data from an Italian community mental health center. Brain Sci..

[7] Mogavero M.P., Lanza G., Bruni O., Ferini-Strambi L., Silvani A., Faraguna U., Ferri R. (2025). Beyond the sleep lab: A narrative review of wearable sleep monitoring. Bioengineering.

[8] Iraniparast M., Shi Y., Wu Y., Zeng L., Maxwell C.J., Kryscio R.J., St John P.D., Santacruz K.S., Tyas S.L. (2022). Cognitive reserve and mild cognitive impairment: Predictors and rates of reversion to intact cognition vs. progression to dementia. Neurology.

[9] Petersen R.C. (2004). Mild cognitive impairment as a diagnostic entity. J. Intern. Med..

[10] Kim J.G., Kim H., Hwang J., Kang S.H., Lee C.N., Woo J., Kim C., Han K., Kim J.B., Park K.W. (2022). Differentiating amnestic from non-amnestic mild cognitive impairment subtypes using graph theoretical measures of electroencephalography. Sci. Rep..

[11] Saleh D., Bertisch S.M., Reid M., Lim A., Purcell S., Redline S. (2025). Actigraphy-derived sleep fragmentation index: Convergent validity and associations with clinical outcomes. J. Clin. Sleep Med..

[12] Liu G.H., Huang Y.H., Yuan T.C., Chen Y.P., Hsu J.L., Lee S.H., Lin C.M., Toh C.H., Fang J.T., Lin S.W. (2025). Associations of wearable-measured sleep and physical activity with memory performance in older adults: Cross-sectional study with actigraphy and MRI. JMIR Aging.

[13] Balouch S., Dijk D.A.D., Rusted J., Skene S.S., Tabet N., Dijk D.J. (2022). Night-to-night variation in sleep associates with day-to-day variation in vigilance, cognition, memory, and behavioral problems in Alzheimer’s disease. Alzheimers Dement..

[14] Batzikosta A., Moraitou D., Steiropoulos P., Masoura E., Papantoniou G., Katsouri I.-G., Sofologi M., Tsentidou G., Tsolaki M. (2025). Sleep trajectories in amnestic and non-amnestic MCI: Longitudinal insights from subjective and objective assessments. Diagnostics.

[15] Cai S., Li T., Zhang L., Shi L., Liao J., Li W., Cheng G., Tan W., Rong S. (2020). Characteristics of sleep structure assessed by objective measurements in patients with amnestic mild cognitive impairment: A meta-analysis. Front. Neurol..

[16] Ho M.K., Saxe R., Cushman F. (2022). Planning with Theory of Mind. Trends Cogn. Sci..

[17] Tsentidou G., Moraitou D., Tsolaki M. (2022). Emotion recognition in a health continuum: Comparison of healthy adults of advancing age, community-dwelling adults bearing vascular risk factors and people diagnosed with mild cognitive impairment. Int. J. Environ. Res. Public Health.

[18] Kessels R.P.C., Elferink M.W., van Tilborg I. (2020). Social cognition and social functioning in patients with amnestic mild cognitive impairment or Alzheimer’s dementia. J. Neuropsychol..

[19] Rothermich K., Giorio C., Falkins S., Leonard L., Roberts A. (2021). Nonliteral language processing across the lifespan. Acta Psychol..

[20] Taylor T., Edwards T.L. (2021). What can we learn by treating perspective taking as problem solving?. Perspect. Behav. Sci..

[21] Long E.L., Catmur C., Fleming S.M., Bird G. (2025). Metacognition facilitates theory of mind through optimal weighting of trait inferences. Cognition.

[22] Bampa G., Moraitou D., Metallidou P., Masoura E., Papantoniou G., Sofologi M., Kougioumtzis G., Papatzikis E., Tsolaki M. (2024). Metacognitive beliefs of efficacy about daily life situations and use of cognitive strategies in amnestic mild cognitive impairment: A cross-sectional study. Front. Psychol..

[23] Chatzidimitriou E., Chen Y., Moraitou D., Ioannidis P., Aretouli E., Kramer J.H., Miller B.L., Gorno-Tempini M., Seeley W.W., Rosen H.J. (2025). The predictive value of social cognition assessment for 1-year follow-up functional outcomes in behavioral variant frontotemporal dementia. J. Neurol..

[24] Alfeo F., Lanciano T., Abbatantuono C., Gintili G., De Caro M.F., Curci A., Taurisano P. (2024). Cognitive, emotional, and daily functioning domains involved in decision-making among patients with mild cognitive impairment: A systematic review. Brain Sci..

[25] Strikwerda-Brown C., Ramanan S., Irish M. (2019). Neurocognitive mechanisms of theory of mind impairment in neurodegeneration: A transdiagnostic approach. Neuropsychiatr. Dis. Treat..

[26] Zimmerman M.E., Benasi G., Hale C., Yeung L.K., Cochran J., Brickman A.M., St-Onge M.P. (2024). The effects of insufficient sleep and adequate sleep on cognitive function in healthy adults. Sleep Health.

[27] Sen A., Tai X.Y. (2023). Sleep duration and executive function in adults. Curr. Neurol. Neurosci. Rep..

[28] Zare F., Shakhmurova G., Rizaev J., Smerat A., Abbood R.S., Patil V., Kumar-Mishra M. (2026). Sleep-dependent clearance of brain metabolites via the glymphatic system: Implications for Alzheimer’s pathophysiology. Brain Behav..

[29] Crowley R., Alderman E., Javadi A.H., Tamminen J. (2024). A systematic and meta-analytic review of the impact of sleep restriction on memory formation. Neurosci. Biobehav. Rev..

[30] Gabb V.G., Blackman J., Morrison H., Li H., Kendrick A., Turner N., Greenwood R., Biswas B., Heslegrave A., Coulthard E. (2025). Longitudinal remote sleep and cognitive research in older adults with mild cognitive impairment and dementia: Prospective feasibility cohort study. JMIR Aging.

[31] Buratti L., Camilletti R., Pulcini A., Rocchi C., Viticchi G., Falsetti L., Baldinelli S., Fiori C., Silvestrini M. (2021). Sleep actigraphic patterns and cognitive status. J. Integr. Neurosci..

[32] Batzikosta A., Moraitou D., Steiropoulos P., Papantoniou G., Kougioumtzis G.A., Katsouri I.-G., Sofologi M., Tsolaki M. (2024). The relationships of specific cognitive control abilities with objective and subjective sleep parameters in mild cognitive impairment: Revealing the association between cognitive planning and sleep duration. Brain Sci..

[33] Batzikosta A., Moraitou D., Steiropoulos P., Papantoniou G., Kougioumtzis G.A., Katsouri I.-G., Sofologi M., Tsolaki M. (2025). Examining specific theory-of-mind aspects in amnestic and non-amnestic mild cognitive impairment: Their relationships with sleep duration and cognitive planning. Brain Sci..

[34] Faul F., Erdfelder E., Lang A.G., Buchner A. (2007). G*Power 3: A flexible statistical power analysis program for the social, behavioral, and biomedical sciences. Behav. Res. Methods.

[35] Petersen R.C., Caracciolo B., Brayne C., Gauthier S., Jelic V., Fratiglioni L. (2014). Mild cognitive impairment: A concept in evolution. J. Intern. Med..

[36] American Psychiatric Association (2022). Diagnostic and Statistical Manual of Mental Disorders.

[37] Yesavage J.A., Brink T.L., Rose T.L., Lum O., Huang V., Adey M., Leirer V.O. (1982). Development and validation of a geriatric depression screening scale: A preliminary report. J. Psychiatr. Res..

[38] Fountoulakis K.N., Tsolaki M., Iacovides A., Yesavage J., O’Hara R., Kazis A., Ierodiakonou C. (1999). The validation of the short form of the geriatric depression scale (GDS) in Greece. Aging Clin. Exp. Res..

[39] Beck A.T., Ward C.H., Mendelson M., Mock J., Erbaugh J. (1961). An inventory for measuring depression. Arch. Gen. Psychiatry.

[40] Beck A.T., Epstein N., Brown G., Steer R.A. (1988). An inventory for measuring clinical anxiety: Psychometric properties. J. Consult. Clin. Psychol..

[41] Sinoff G., Ore L., Zlotogorsky D., Tamir A. (1999). Short anxiety screening test—A brief instrument for detecting anxiety in the elderly. Int. J. Geriatr. Psychiatry.

[42] Grammatikopoulos I.A., Sinoff G., Alegakis A., Kounalakis D., Antonopoulou M., Lionis C. (2010). The short anxiety screening test in Greek: Translation and validation. Ann. Gen. Psychiatry.

[43] Cummings J.L., Mega M., Gray K., Rosenberg-Thompson S., Carusi D.A., Gornbein J. (1994). The neuropsychiatric inventory: Comprehensive assessment of psychopathology in dementia. Neurology.

[44] Politis A.M., Mayer L.S., Passa M., Maillis A., Lyketsos C.G. (2004). Validity and reliability of the newly translated Hellenic Neuropsychiatric Inventory (H-NPI) applied to Greek outpatients with Alzheimer’s disease. Int. J. Geriatr. Psychiatry.

[45] Folstein M.F., Folstein S.E., McHugh P.R. (1975). Mini-mental state: A practical method for grading the cognitive state of patients for the clinician. J. Psychiatr. Res..

[46] Fountoulakis K.N., Tsolaki M., Chantzi H., Kazis A.D. (2000). Mini Mental State Examination (MMSE): A validation study in Greece. Am. J. Alzheimers Dis. Other Demen..

[47] Nasreddine Z.S., Phillips N.A., Bédirian V., Charbonneau S., Whitehead V., Collin I., Cummings J.L., Chertkow H. (2005). The Montreal Cognitive Assessment (MoCA): A brief screening tool for mild cognitive impairment. J. Am. Geriatr. Soc..

[48] Poptsi E., Moraitou D., Eleftheriou M., Kounti-Zafeiropoulou F., Papasozomenou C., Agogiatou C., Bakoglidou E., Batsila G., Liapi D., Markou N. (2019). Normative data for the Montreal Cognitive Assessment in Greek older adults with subjective cognitive decline, mild cognitive impairment, and dementia. J. Geriatr. Psychiatry Neurol..

[49] Kounti F., Tsolaki M., Kiosseoglou G. (2006). Functional Cognitive Assessment Scale (FUCAS): A new scale to assess executive cognitive function in daily life activities in patients with dementia and mild cognitive impairment. Hum. Psychopharmacol..

[50] Reisberg B., Ferris S.H., de Leon M.J., Crook T. (1982). The Global Deterioration Scale for assessment of primary degenerative dementia. Am. J. Psychiatry.

[51] Tsolaki M., Poptsi E., Aggogiatou C., Markou N., Zafeiropoulos S. (2017). Computer-based cognitive training versus paper and pencil training: Which is more effective? A randomized controlled trial in people with mild cognitive impairment. JSM Alzheimers Dis. Relat. Dement..

[52] Yuan H., Hill E.A., Kyle S.D., Doherty A. (2024). A systematic review of the performance of actigraphy in measuring sleep stages. J. Sleep Res..

[53] Lee M.P., Hoang K., Park S., Song Y.M., Joo E.Y., Chang W., Kim J.H., Kim J.K. (2024). Imputing missing sleep data from wearables with neural networks in real-world settings. Sleep.

[54] Chinoy E.D., Cuellar J.A., Huwa K.E., Jameson J.T., Watson C.H., Bessman S.C., Hirsch D.A., Cooper A.D., Drummond S.P.A., Markwald R.R. (2021). Performance of seven consumer sleep-tracking devices compared with polysomnography. Sleep.

[55] Delis D.C., Kaplan E., Kramer J.H. (2001). Delis-Kaplan Executive Function System (D-KEFS).

[56] Corbo I., Troisi G., Marselli G., Casagrande M. (2024). The role of cognitive flexibility on higher level executive functions in mild cognitive impairment and healthy older adults. BMC Psychol..

[57] Hessen E. (2025). Mild Cognitive Impairment and Neuropsychological Examination. Front. Psychol..

[58] Tsatali M., Surdu F., Konstantinou A., Moraitou D. (2024). Normative Data for the D-KEFS Color-Word Interference and Trail Making Tests Adapted in Greek Adult Population 20–49 Years Old. NeuroSci.

[59] Natsopoulos D. Theory of Mind Battery.

[60] Nazlidou E.I., Moraitou D., Natsopoulos D., Papantoniou G. (2015). Social cognition in adults: The role of cognitive control. Hell. J. Nucl. Med..

[61] Nazlidou E., Moraitou D., Natsopoulos D., Papaliagkas V., Masoura E., Papantoniou G. (2018). Inefficient understanding of non-factive mental verbs with social aspect in adults: Comparison to cognitive factive verb processing. Neuropsychiatr. Dis. Treat..

[62] McDonald S., Bornhofen C., Shum D.H.K., Long E., Saunders C., Neulinger K. (2006). Reliability and Validity of The Awareness of Social Inference Test (TASIT): A Clinical Test of Social Perception. Disabil. Rehabil..

[63] Moraitou D., Papantoniou G., Gkinopoulos T., Nigritinou M. (2013). Older Adults’ Decoding of Emotions: Age-Related Differences in Interpreting Dynamic Emotional Displays and the Well-Preserved Ability to Recognize Happiness. Psychogeriatrics.

[64] IBM Corp (2022). IBM SPSS Statistics for Windows.

[65] The jamovi Project (2024). jamovi.

[66] Kline R.B. (2016). Principles and Practice of Structural Equation Modeling.

[67] Brown T.A. (2006). Confirmatory Factor Analysis for Applied Research.

[68] Hu L., Bentler P.M. (1999). Cutoff criteria for fit indexes in covariance structure analysis: Conventional criteria versus new alternatives. Struct. Equ. Model..

[69] Kenny D.A., Kaniskan B., McCoach D.B. (2015). The performance of RMSEA in models with small degrees of freedom. Sociol. Methods Res..

[70] Shi D., Lee T., Maydeu-Olivares A. (2022). Evaluating SEM Model Fit with Small Degrees of Freedom. Multivar. Behav. Res..

[71] Cipriani G.E., Molfese S., Giovannelli F., Güntekin B., Vitali N., Marcato R., Amanzio M. (2025). Executive control from healthy ageing to cognitive impairment: A systematic review of Stroop and Simon effects using psychophysiological and imaging techniques. Neurosci. Biobehav. Rev..

[72] Rodrigues E.A., Christie G.J., Cosco T., Farzan F., Sixsmith A., Moreno S. (2024). A subtype perspective on cognitive trajectories in healthy aging. Brain Sci..

[73] Stern Y., Arenaza-Urquijo E.M., Bartrés-Faz D., Belleville S., Cantilon M., Chételat G., Ewers M., Franzmeier N., Kempermann G., Kremen W.S. (2020). Reserve, resilience and protective factors in aging and Alzheimer’s disease. Alzheimer’s Dement..

[74] Abrous D.N., Blin N., Boraxbekk C.-J., Catheline G., Fitzsimons C.P., Hilscher M., Lemoine M., Lopes L.V., Maass A., Nilsson M. (2026). Hallmarks of Healthy Cognitive Aging: Inter-Individual Differences in Aging Trajectories. Ageing Res. Rev..

[75] Che J., Cheng N., Jiang B., Liu Y., Liu H., Li Y., Liu H. (2024). Executive Function Measures of Participants with Mild Cognitive Impairment: Systematic Review and Meta-Analysis of Event-Related Potential Studies. Int. J. Psychophysiol..

[76] Pantsiou K., Sfakianaki O., Papaliagkas V., Savvoulidou D., Costa V., Papantoniou G., Moraitou D. (2018). Inhibitory control, task/rule switching, and cognitive planning in vascular dementia: Are there any differences from vascular aging?. Front. Aging Neurosci..

[77] Rodríguez-Nieto G., Mercadillo R.E., Pasaye E.H., Barrios F.A. (2022). Affective and cognitive brain-networks are differently integrated in women and men while experiencing compassion. Front. Psychol..

[78] Chino B., Torres-Simón L., Żelwetro A., Rodríguez-Rojo I.C., Carnes-Vendrell A., Piñol-Ripoll G., Yubero R., Paúl N., Maestú F. (2023). Understanding the episodic memory and executive functioning axis impairment in MCI patients: A multicenter study in comparison with CSF biomarkers. Biomedicines.

[79] Menon V., D’Esposito M. (2022). The Role of PFC Networks in Cognitive Control and Executive Function. Neuropsychopharmacology.

[80] Wei L., Lu J., Li X., Yang H., Wang H., Zhu Z., Chen J., Zhang B. (2026). Subtype-specific brain atrophy and white matter alterations in mild cognitive impairment. Brain Sci..

[81] Csukly G., Sirály E., Fodor Z., Horváth A., Salacz P., Hidasi Z., Csibri É., Rudas G., Szabó Á. (2016). The differentiation of amnestic type MCI from the non-amnestic types by structural MRI. Front. Aging Neurosci..

[82] Gross R.G., Grossman M. (2010). Executive Resources. Continuum.

[83] Henry J.D., von Hippel W., Molenberghs P., Lee T., Sachdev P.S. (2016). Clinical assessment of social cognitive function in neurological disorders. Nat. Rev. Neurol..

[84] Morellini L., Izzo A., Ceroni M., Rossi S., Zerboni G., Rege-Colet L., Biglia E., Sacco L. (2022). Theory of mind in patients with mild cognitive impairment: A systematic review. Front. Psychol..

[85] Roheger M., Brenning J., Riemann S., Martin A.K., Flöel A., Meinzer M. (2022). Progression of Socio-Cognitive Impairment from Healthy Aging to Alzheimer’s Dementia: A Systematic Review and Meta-Analysis. Neurosci. Biobehav. Rev..

[86] Eramudugolla R., Huynh K., Zhou S., Amos J.G., Anstey K.J. (2022). Social cognition and social functioning in MCI and dementia in an epidemiological sample. J. Int. Neuropsychol. Soc..

[87] Hauptman M., Blank I., Fedorenko E. (2023). Non-literal language processing is jointly supported by the language and theory of mind networks: Evidence from a novel meta-analytic fMRI approach. Cortex.

[88] Delage E., Rouleau I., Akzam-Ouellette M.A., Roy-Côté F., Joubert S., CIMA-Q (2024). An examination of semantic performance in mild cognitive impairment progressors and nonprogressors. Neuropsychology.

[89] Schurz M., Radua J., Tholen M.G., Maliske L., Margulies D.S., Mars R.B., Sallet J., Kanske P. (2021). Toward a hierarchical model of social cognition: A neuroimaging meta-analysis and integrative review of empathy and theory of mind. Psychol. Bull..

[90] Michaelian J.C., Mowszowski L., Guastella A.J., Henry J.D., Duffy S., McCade D., Naismith S.L. (2019). Theory of mind in mild cognitive impairment—Relationship with limbic structures and behavioural change. J. Int. Neuropsychol. Soc..

[91] Song J. (2023). Amygdala activity and amygdala–hippocampus connectivity: Metabolic diseases, dementia, and neuropsychiatric issues. Biomed. Pharmacother..

[92] Yang J., Huang L. (2023). Comprehension of metaphors in patients with mild cognitive impairment: Evidence from behavioral and ERP data. Acta Psychol..

[93] Kljajevic V. (2022). Older and wiser: Interpretation of proverbs in the face of age-related cortical atrophy. Front. Aging Neurosci..

[94] Dimitriou N.K., Folia V., Nousia A., Messinis L., Nasios G. (2026). Effects of cognitive and language decline on communication in mild cognitive impairment: An integrative systematic review. Appl. Neuropsychol. Adult.

[95] Benkirane O., Simor P., Mairesse O., Peigneux P. (2024). Sleep Fragmentation Modulates the Neurophysiological Correlates of Cognitive Fatigue. Clocks Sleep.

[96] Buxton O.M., Gao Q., Hakun J.G., Ji L., Gamaldo A.A., Bertisch S.M., Sliwinski M.J., Wang C., Derby C.A. (2026). Within- and Between-Person Associations of Sleep Characteristics with Daily Cognitive Performance in a Community-Based Sample of Older Adults. Sleep Health.

[97] Ying F., Wen J.H., Klaiber P., DeLongis A., Slavish D.C., Sin N.L. (2022). Associations Between Intraindividual Variability in Sleep and Daily Positive Affect. Affect. Sci..

[98] Tai X.Y., Chen C., Manohar S., Husain M. (2022). Impact of Sleep Duration on Executive Function and Brain Structure. Commun. Biol..

[99] Babasoy Y. (2025). Cognitive Pragmatics: Mental Processes Underlying Contextual Meaning Construction. Glob. Spectr. Res. Humanit..

[100] Boccadoro S., Cracco E., Hudson A.R., Bardi L., Nijhof A.D., Wiersema J.R., Brass M., Mueller S.C. (2019). Defining the Neural Correlates of Spontaneous Theory of Mind (ToM): An fMRI Multi-Study Investigation. NeuroImage.

[101] Arioli M., Crespi C., Canessa N. (2018). Social Cognition through the Lens of Cognitive and Clinical Neuroscience. Biomed. Res. Int..

[102] Lucena A.T.D., Bhalla R.K., Belfort Almeida Dos Santos T.T., Dourado M.C.N. (2020). The relationship between theory of mind and cognition in Alzheimer’s disease: A systematic review. J. Clin. Exp. Neuropsychol..

[103] Cudney L.E., Frey B.N., McCabe R.E., Green S.M. (2022). Investigating the Relationship between Objective Measures of Sleep and Self-Report Sleep Quality in Healthy Adults: A Review. J. Clin. Sleep Med..

[104] Krause A.J., Simon E.B., Mander B.A., Winer J.R., Walker M.P. (2020). The Sleep-Deprived Human Brain. Nat. Rev. Neurosci..

[105] Yu W., Chen J., Kong Z., Sun W., Zhou X., Lu L., Gao X., Sun H. (2024). Understanding the Cognitive and Neuroimaging Bases Underlying the Impact of Sleep Deprivation on Social Function. iScience.

[106] Martiadis V., Pessina E., Raffone F., Iniziato V., Martini A., Scognamiglio P. (2023). Metacognition in Schizophrenia: A Practical Overview of Psychometric Metacognition Assessment Tools for Researchers and Clinicians. Front. Psychiatry.

[107] Beattie L., Kyle S.D., Espie C.A., Biello S.M. (2015). Social Interactions, Emotion and Sleep: A Systematic Review and Research Agenda. Sleep Med. Rev..

[108] Palmer C.A., Alfano C.A. (2017). Sleep and Emotion Regulation: An Organizing, Integrative Review. Sleep Med. Rev..

[109] Ben Simon E., Rossi A., Harvey A.G., Walker M.P. (2020). Overanxious and Underslept. Nat. Hum. Behav..

[110] Gordon A.M., Chen S. (2014). The Role of Sleep in Interpersonal Conflict: Do Sleepless Nights Mean Worse Fights?. Soc. Psychol. Personal. Sci..

[111] Bottenheft C., Hogenelst K., Stuldreher I., Kleemann R., Groen E., van Erp J., Brouwer A.M. (2023). Understanding the Combined Effects of Sleep Deprivation and Acute Social Stress on Cognitive Performance Using a Comprehensive Approach. Brain Behav. Immun. Health.

[112] Torossian M., Fiske S.M., Jacelon C.S. (2021). Sleep, Mild Cognitive Impairment, and Interventions for Sleep Improvement: An Integrative Review. West. J. Nurs. Res..

