# Supplementary material for: Longitudinal Trajectories of Cognitive Control and Theory of Mind in Amnestic and Non-Amnestic Mild Cognitive Impairment: Prospective Associations of Objective Sleep Duration
Source: J Clin Med. 2026 Jul 23;15(15):5780. doi: 10.3390/jcm15155780 (PMC13467327; doi:10.3390/jcm15155780)
Supplement: Supplementary file 1 [file jcm-15-05780-s001.zip › jcm-4385083-supplementary.pdf]

Supplementary Materials for: Longitudinal Trajectories of Cognitive Control and Theory of Mind in Amnestic and Non-Amnestic Mild Cognitive Impairment: Prospective Effects of Objective Sleep Duration

Supplementary Table S1

Correlations Between Sleep Parameters and Cognitive Control Performance Across the three Assessments

**Variable**	TST (T1)	TST (T2)	TST (T3)	SE (T1)	SE (T2)	SE (T3)	WASO (T1)	WASO (T2)	WASO (T3)
TT: Total achievement score (T1)	.157*	.309**	.306**	-	-	-	.159*	-	-
TT: Total achievement score (T2)	.173*	.343**	.344**	-	-	-	-	-	-
TT: Total achievement score (T3)	.170*	.343**	.344**	-	-	-	-	-	-
TT: Total number of problems (T1)	.171*	.295**	.289**	-	-	-	-	-	-
TT: Total number of problems (T2)	.204**	.336**	.331**	-	-	-	-	-	-
TT: Total number of problems (T3)	.210**	.359**	.354**	-	-	-	-	-	-
TT: Total Number of Rule Violations (T1)	-.216**	-.336**	-.333**	-	-.149*	-.180*	-	-	-
TT: Total Number of Rule Violations (T2)	-.217**	-.345**	-.340**	-	-.152*	-.184*	-	-	-
TT: Total Number of Rule Violations (T3)	-.210**	-.341**	-.336**	-	-.166*	-.197*	-	-	-
VFT: Condition (IIIa) (T1)	-	-	-	-	-	-	-	-	-
VFT: Condition (IIIa) (T2)	-	.175*	.177*	-	-	-	-	-	-
VFT: Condition (IIIa) (T3)	-	.207**	.217**	-	-	-	-	-	-
VFT: Condition (IIIb) (T1)	-	-	-	-	-	-	-	-	-
VFT: Condition (IIIb) (T2)	-	-	-	-	-	-	-	-	-
VFT: Condition (IIIb) (T3)	-	.153*	.157*	-	-	-	-	-	-
CWIT: Condition (III) (T1)	-	-	-	-	-	-	-	-	-
CWIT: Condition (III) (T2)	-	-	-	-	-	-	-	-	-
CWIT: Condition (III) (T3)	-	-	-	-	-	-	-	-	-
CWIT: Condition (IV) (T1)	-	-	-	-	-	-	-	-	-
CWIT: Condition (IV) (T2)	-	-	-	-	-	-	-	-	-
CWIT: Condition (IV) (T3)	-	-	-	-	-	-	-	-	-

Note: TT: Tower Test; VFT: Verbal Fluency Test; CWIT: Color–Word Interference Test. Values are Pearson correlation coefficients (r). N = 179. * $p < .05$, ** $p < .01$.

Supplementary Table S2

Correlations Between Sleep Parameters and ToM Performance Across the three Assessments.

**Variable**	TST (T1)	TST (T2)	TST (T3)	SE (T1)	SE (T2)	SE (T3)	WASO (T1)	WASO (T2)	WASO (T3)
Non-literal speech 1 (T1)	-	.205**	.204**	-	.148*	.174*	-		-.157*
Non-literal speech 1 (T2)	-	.246**	.247**	-	.188*	.213**	-	-.148*	-.163*
Non-literal speech 1 (T3)	.187*	.328**	.333**	-	.259**	.296**	-	-.081*	-.194**
Non-literal speech 2 (T1)	.186*	.318**	.321**	-	.252**	.290**	-	-.158*	-.163*
Non-literal speech 2 (T2)	.178*	.308**	.310**	-	.254**	.288**	-	-.147*	-.152*
Non-literal speech 2 (T3)	.221**	.385**	.385**	-	.252**	.291**	-	-	-
Verbal Metaphors in Context (T1)	-	-	-	-	-	-	.157*	.152*	-
Verbal Metaphors in Context (T2)	-	-	-	-	-	-	.165*	.152*	-
Verbal Metaphors in Context (T3)	-	.226**	.237**	-	-	-	-	-	-
Nominal Metaphors in Context (T1)	-	-	-	-	-	-	-	-	-
Nominal Metaphors in Context (T2)	-	.170*	.175*	-	-	-	-	-	-
Nominal Metaphors in Context (T3)	-	.197**	.201**	-	-	-	-	-	-
Proverbs in Context (T1)	.193**	.280**	.273**	-	.159*	.185*	-	-.139	-.150*
Proverbs in Context (T2)	.171*	.267**	.254**	-	.185*	.209**	-	-.160*	-.167*
Proverbs in Context (T3)	.174**	.310**	.295**	-	.158*	.188*	-	-	-
Third order ToM stories (T1)	.237**	.376**	.369**	-	.213**	.242**	-	-	-
Third order ToM stories (T2)	.222**	.368**	.351**	-	.203**	.232**	-	-.150*	-
Third order ToM stories (T3)	.247**	.391**	.383**	-	.212**	.250**	-	-	-
TASIT-PART 1 (Neutral) (T1)	-	.147*	.160*	-	-	-	-	-	-
TASIT-PART 1 (Neutral) (T2)	-	.211**	.223**	-	-	-	-	-	-
TASIT-PART 1 (Neutral) (T3)	-	-	.170*	-	-	-	-	-	-
TASIT-PART 1 (Happy) (T1)	-	.162*	.161*	-	-	-	-	-	-
TASIT-PART 1 (Happy) (T2)	.227**	.301**	.296**	-	.158*	.189**	-	-	-
TASIT-PART 1 (Happy) (T3)	.165*	.261**	.240**	-	-	.155*	-	-	-
TASIT-PART 1 (Decoding of uncertain emotional expressions) (T1)	.168*	.249**	.265**	-	-	-	-	-	-
TASIT-PART 1 (Decoding of uncertain emotional expressions) (T2)	.211**	.298**	.310**	-	-	-	-	-	-
TASIT-PART 1 (Decoding of uncertain emotional expressions) (T3)	.249**	.356**	.364**	-			-	-	-

Note. Values are Pearson correlation coefficients (r). N = 179. * p < .05, ** p < .01.

Supplementary Table S3

Correlations Between Theory of Mind Variables and Tower Test Across the Three Assessments

**Variable**	TT: Total achievement score (T1)	TT: Total achievement score (T2)	TT: Total achievement score (T3)	TT: Total number of problems (T1)	TT: Total number of problems (T2)	TT: Total number of problems (T3)	TT: Total Number of Rule Violations (T1)	TT: Total Number of Rule Violations (T2)	TT: Total Number of Rule Violations (T3)
Non-literal speech 1 (T1)	.183*	.276**	.261**	.293**	.361**	.382**	-.428**	-.417**	-.425**
Non-literal speech 1 (T2)	.207**	.299**	.295**	.333**	.389**	.425**	-.450**	-.445**	-.459**
Non-literal speech 1 (T3)	.299**	.393**	.371**	.402**	.470**	.494**	-.515**	-.527**	-.538**
Non-literal speech 2 (T1)	.321**	.416**	.402**	.407**	.490**	.521**	-.553**	-.571**	-.591**
Non-literal speech 2 (T2)	.300**	.396**	.390**	.400**	.478**	.514**	-.536**	-.556**	-.572**
Non-literal speech 2 (T3)	.387**	.481**	.483**	.473**	.541**	.580**	-.534**	-.549**	-.566**
Verbal Metaphors in Context (T1)	.276**	.302**	.302**	.289**	.317**	.321**	-	-	-
Verbal Metaphors in Context (T2)	.263**	.306**	.296**	.274**	.318**	.313**	-.197**	-.195**	-.200**
Verbal Metaphors in Context (T3)	.390**	.456**	.452**	.355**	.419**	.465**	-.385**	-.289**	-.417**
Nominal Metaphors in Context (T1)	.284**	.329**	.315**	.285**	.301**	.358**	-.269**	-.275**	-.300**
Nominal Metaphors in Context (T2)	.321**	.368**	.356**	.322**	.337**	.395**	-.269**	-.275**	-.283**
Nominal Metaphors in Context (T3)	.326**	.398**	.404**	.345**	.393**	.481**	-.367**	-.383**	-.403**
Proverbs in Context (T1)	.255**	.323**	.321**	.306**	.365**	.390**	-.315**	-.334**	-.338**
Proverbs in Context (T2)	.246**	.321**	.330**	.285**	.349**	.390**	-.384**	-.401**	-.417**
Proverbs in Context (T3)	3.14**	.397**	.406**	.361**	.425**	.476**	-.430**	-.445**	-.473**
Third order ToM stories (T1)	.383**	.452**	.430**	.379**	.463**	.508**	-.505**	-.519**	-.534**
Third order ToM stories (T2)	.374**	.445**	.426**	.393**	.474**	.519**	-.473**	-.487**	-.505**
Third order ToM stories (T3)	.410**	.489**	.477**	.407**	.486**	.535**	-.551**	-.567**	-.583**
TASIT-PART 1 (Neutral) (T1)	.235**	.261**	.269**	.297**	.319**	.329**	-.194**	-.200**	-.205**

TASIT-PART 1 (Neutral) (T2)	.324**	.353**	.331**	.377**	.421**	.424**	-.230**	-.233**	-.244**
TASIT-PART 1 (Neutral) (T3)	.413**	.438**	.403**	.324**	.346**	.364**	-.320**	-.326**	-.331**
TASIT-PART 1 (Happy) (T1)	-	-	-	-	-	-	-	-	-
TASIT-PART 1 (Happy) (T2)	.227**	.266**	.238**	.242*	.289**	.300**	-.234**	-.255**	-.254**
TASIT-PART 1 (Happy) (T3)	.228**	.262**	.219**	.267**	.308**	.278**	-.284**	-.293**	-.307**
TASIT-PART 1 (Decoding of uncertain emotional expressions) (T1)	.341**	.388**	.364**	.341**	.400**	.410**	-.308**	-.314**	-.348**
TASIT-PART 1 (Decoding of uncertain emotional expressions) (T2)	.361**	.414**	.384**	.366**	.429**	.440**	-.329**	-.338**	-.372**
TASIT-PART 1 (Decoding of uncertain emotional expressions) (T3)	.330**	.393**	.382**	.384**	.448**	.440**	-.446**	-.450**	-.481**

Note. Values are Pearson correlation coefficients (r). N = 179. * $p < .05$, ** $p < .01$.

Supplementary Table S4

Correlations Between Theory of Mind Variables and Verbal Fluency Test Across the Three Assessments

**Variable**	VFT: Condition (IIIa) (T1)	VFT: Condition (IIIa) (T2)	VFT: Condition (IIIa) (T3)	VFT: Condition (IIIb) (T1)	VFT: Condition (IIIb) (T2)	VFT: Condition (IIIb) (T3)
Non-literal speech 1 (T1)	-	-	-	-	-	-
Non-literal speech 1 (T2)	-	-	.169*	-	-	-
Non-literal speech 1 (T3)	-	.244**	.278**	-	.169*	.201**
Non-literal speech 2 (T1)	-	.219**	.251**	-	-	.151*
Non-literal speech 2 (T2)	-	.208**	.240**	-	-	.147*
Non-literal speech 2 (T3)	-	.285*	.324	-	.200**	.231**
Verbal Metaphors in Context (T1)	-	-	.150*	-	.164*	.193**
Verbal Metaphors in Context (T2)	-	.169*	.194**	-	.181*	.200**
Verbal Metaphors in Context (T3)	-	.289**	.333**	-	.253**	.287**
Nominal Metaphors in Context (T1)	-	.293**	.274**	-	.231**	.226**
Nominal Metaphors in Context (T2)	.172*	.319**	.317**	-	.260**	.265**
Nominal Metaphors in Context (T3)	.151*	.351**	.374**	-	.296**	.321**
Proverbs in Context (T1)	-	.163**	.205**	-	-	.166*
Proverbs in Context (T2)	-	.200*	.260**	-	.166*	.203**
Proverbs in Context (T3)	-	.256**				
Third order ToM stories (T1)	-	.280**	-.505**	-	-	-
Third order ToM stories (T2)	-	.302**	.348**	-	.252**	.283**
Third order ToM stories (T3)	-	.311**	.366**	-	.246**	.284**
TASIT-PART 1 (Neutral) (T1)	-	-	-	-	-	-
TASIT-PART 1 (Neutral) (T2)	-	-	.147*	-	-	-
TASIT-PART 1 (Neutral) (T3)	-	.211**	.247**	-	.202**	.216**
TASIT-PART 1 (Happy) (T1)	-	-	-	-	-	-
TASIT-PART 1 (Happy) (T2)	-	.219**	.272**	-	.161*	.202**
TASIT-PART 1 (Happy) (T3)	-	.195**	.254**	-	-	.183*
TASIT-PART 1 (Decoding of uncertain emotional expressions) (T1)	-	.259**	.291**	-	.157*	.170*
TASIT-PART 1 (Decoding of uncertain emotional expressions) (T2)	-	.287**	.326**	-	.186*	.204**

TASIT-PART 1 (Decoding of uncertain emotional expressions) (T3)	-	.289**	.338**	-	.197**	.223**

Note. Values are Pearson correlation coefficients (r). N = 179. * p < .05, ** p < .01.

Supplementary Table S5

Correlations Between Theory of Mind Variables and Color–Word Interference Test Across the Three Assessments

**Variable**	CWIT: Condition (III) (T1)	CWIT: Condition (III) (T2)	CWIT: Condition (III) (T3)	CWIT: Condition (IV) (T1)	CWIT: Condition (IV) (T2)	CWIT: Condition (IV) (T3)
Non-literal speech 1 (T1)	-	-	-	-.151*	-.199*	-.196**
Non-literal speech 1 (T2)	-	-	-	-	-.168*	-.172*
Non-literal speech 1 (T3)	-	-	-	-	-	-
Non-literal speech 2 (T1)	-	-	-.210**	-	-.152*	-.185*
Non-literal speech 2 (T2)	-	-	-.188*	-	-.163*	-.184*
Non-literal speech 2 (T3)	-	-	-.155*	-	-.168*	-.174*
Verbal Metaphors in Context (T1)	-	-	-.235**	-.199**	-.214**	-.273**
Verbal Metaphors in Context (T2)	-	-	-.153*	-.148*	-.159*	-.208**
Verbal Metaphors in Context (T3)	-.161*	-.220**	-.236**	-	-.161*	-.195**
Nominal Metaphors in Context (T1)	-.148*	-.194**	-.239**	-.157*	-.207**	-.243**
Nominal Metaphors in Context (T2)	-.201**	-.240**	-.263**	-.210*	-.260**	-.278**
Nominal Metaphors in Context (T3)	-.164*	-.264**	-.291**	-	-.224**	-.245**
Proverbs in Context (T1)	-	-	-.162*	-	-.202**	-.236**
Proverbs in Context (T2)	-	-.182*	-.218*	-	-.182*	-.2.16**
Proverbs in Context (T3)	-	-.198**	-.248**	-	-.166*	-.200**
Third order ToM stories (T1)	-	-.165*	-.254**	-	-.186*	-.228**
Third order ToM stories (T2)	-	-.154*	-.240**	-	-.156*	-.197**
Third order ToM stories (T3)	-	-.178*	-.298**	-	-	-.197**
TASIT-PART 1 (Neutral) (T1)	-	-.170*	-	-	-	-
TASIT-PART 1 (Neutral) (T2)	-	-	-	-	-	-
TASIT-PART 1 (Neutral) (T3)	-	-	-.152*	-	-	-
TASIT-PART 1 (Happy) (T1)	-	-	-	-	-	-
TASIT-PART 1 (Happy) (T2)	-	-	-	-	-	-
TASIT-PART 1 (Happy) (T3)	-	-	-	-	-	-

TASIT-PART 1 (Decoding of uncertain emotional expressions) (T1)	-	-	-	-	-	-
TASIT-PART 1 (Decoding of uncertain emotional expressions) (T2)	-	-.166*	-.168*	-	-	-
TASIT-PART 1 (Decoding of uncertain emotional expressions) (T3)	-	-.187*	-.200**	-	-	-

Note. Values are Pearson correlation coefficients (r). N = 179. * p < .05, ** p < .01.

Table S6. Fit indices for all longitudinal path models examining the associations between objective sleep parameters and cognitive control outcomes.

Sleep Parameter	Outcome	χ^2(df)	CFI	RMSE	SRMR
SE	TT: Total achievement score	57.6 (7)	.976	.201	.065
	TT: Total number of problems	42.1 (7)	.982	.167	.058
	TT: Total Number of Rule Violations	41.4 (7)	.986	.166	.061
	VFT: Condition (IIIa)	65.9 (7)	.965	.217	.038
	VFT: Condition (IIIb)	66.1 (7)	.970	.217	.027
	CWIT: Condition (III)	24.9 (7)	.988	.119	.017
	CWIT: Condition (IV)	23.7 (7)	.990	.115	.016
WASO	TT: Total achievement score	44.0 (7)	.984	.172	.027
	TT: Total number of problems	34.0 (7)	.988	.147	.022
	TT: Total Number of Rule Violations	32.6 (7)	.990	.143	.024
	VFT: Condition (IIIa)	52.3 (7)	.976	.190	.016
	VFT: Condition (IIIb)	53.5 (7)	.979	.193	.010
	CWIT: Condition (III)	22.1 (7)	.991	.110	.015
	CWIT: Condition (IV)	30.6 (7)	.988	.137	.009
TST	TT: Total achievement score	58.0 (7)	.983	.202	.085
	TT: Total number of problems	36.1 (7)	.984	.152	.068
	TT: Total Number of Rule Violations	34.6 (7)	.987	.148	.071
	VFT: Condition (IIIa)	60.9 (7)	.965	.207	.055
	VFT: Condition (IIIb)	59.8 (7)	.971	.205	.045
	CWIT: Condition (III)	21.1 (7)	.989	.106	.023
	CWIT: Condition (IV)	26.8 (7)	.987	.126	.018

Note. *χ^2 = chi-square goodness-of-fit statistic; CFI = Comparative Fit Index; RMSEA = Root Mean Square Error of Approximation; SRMR = Standardized Root Mean Square Residual.*

Table S7. Standardized prospective path coefficients for all longitudinal path models examining the associations between objective sleep parameters and cognitive outcomes.

Sleep Parameter	Outcome	Path	β	SE	95% CI	p
SE	TT: Total achievement score	T1→T2	−.002	.006	[−.010, .012]	.893
		T2→T3	−.01	.009	[−.020, .013]	.702
	TT: Total number of problems	T1→T2	.01	.003	[−.005, .008]	.629
		T2→T3	.03	.004	[−.003, .013]	.201

	TT: Total Number of Rule Violations	T1→T2	.00	.006	[−.010, .012]	.889
		T2→T3	−.02	.006	[−.021, .004]	.175
	VFT: Condition (IIIa)	T1→T2	.035	.006	[−.005, .017]	.309
		T2→T3	−.005	.005	[−.010, .009]	.860
	VFT: Condition (IIIb)	T1→T2	.006	.012	[−.021, .027]	.809
		T2→T3	.003	.009	[−.016, .019]	.882
	CWIT: Condition (III)	T1→T2	.022	.007	[−.009, .016]	.596
		T2→T3	−.020	.008	[−.020, .012]	.637
	CWIT: Condition (IV)	T1→T2	−.002	.006	[−.013, .012]	.953
		T2→T3	−.043	.008	[−.025, .005]	.184
WASO	TT: Total achievement score	T1→T2	−.010	.001	[−.003, .001]	.485
		T2→T3	.001	.002	[−.003, .001]	.983
	TT: Total number of problems	T1→T2	−.002	.001	[−.001, .001]	.918
		T2→T3	−.035	.001	[−.003, .000]	.121
	TT: Total Number of Rule Violations	T1→T2	−.001	.001	[−.002, .001]	.955
		T2→T3	-.009	.001	[−.002, .001]	.486
	VFT: Condition (IIIa)	T1→T2	−.028	.001	[−.003, .001]	.421
		T2→T3	.024	.001	[−.001, .003]	.392
	VFT: Condition (IIIb)	T1→T2	-.008	.002	[−.005, .004]	.756
		T2→T3	.012	.002	[−.002, .004]	.501
	CWIT: Condition (III)	T1→T2	−.034	.001	[−.003, .001]	.403
		T2→T3	−.017	.002	[−.004, .002]	.680
	CWIT: Condition (IV)	T1→T2	−.004	.001	[−.002, .002]	.897
		T2→T3	.002	.002	[−.003, .003]	.948
TST	TT: Total achievement score	T1→T2	.020	.001	[−.000, .003]	.172
		T2→T3	.015	.001	[−.001, .002]	.487
	**TT: Total number of problems**	**T1→T2**	**.039**	**.001**	**[.000, .003]**	**.032**
		T2→T3	.044	.001	[−.000, .002]	.055
	TT: Total Number of Rule Violations	T1→T2	−.001	.001	[−.002, .001]	.955
		T2→T3	−.009	.001	[−.002, .001]	.486
	**VFT: Condition (IIIa)**	**T1→T2**	**.071**	**.001**	**[.000, .003]**	**.037**
		T2→T3	.052	.001	[−.000, .002]	.062
	VFT: Condition (IIIb)	T1→T2	.032	.002	[−.001, .005]	.204
		T2→T3	.029	.001	[−.000, .004]	.102
	CWIT: Condition (III)	T1→T2	.014	.001	[−.001, .002]	.729
		T2→T3	−.023	.001	[−.003, .001]	.582
	CWIT: Condition (IV)	T1→T2	−.012	.001	[−.002, .001]	.670
		T2→T3	−.022	.001	[−.003, .001]	.492

Note. β = standardized path coefficient; SE = standard error; CI = confidence interval; T1 = baseline; T2 = first follow-up; T3 = second follow-up. Only prospective cross-lagged paths are presented; autoregressive paths and residual covariances are omitted for brevity.

Table S8. Model fit indices for the longitudinal path models examining the associations between objective sleep parameters and ToM outcomes.

**Sleep Parameter**	**Outcome**	**χ^2(df)**	**CFI**	**RMSE**	**SRMR**

SE	Non-literal speech 1	28.3 (7)	.987	.130	.049
	Non-literal speech 2	59.1 (7)	.972	.204	.071
	Verbal Metaphors in Context	34.2 (7)	.981	.147	.026
	Nominal Metaphors in Context	37.8 (7)	.981	.157	.045
	Proverbs in Context	38.8 (7)	.983	.159	0.53
	Third order ToM stories	43.4 (7)	.979	.170	.062
	TASIT-PART 1 (Neutral)	20.2 (7)	.990	.102	.032
	TASIT-PART 1 (Happy)	25.3 (7)	.985	.121	.038
	TASIT-PART 1 (Decoding of uncertain emotional expressions)	30.8 (7)	.987	.138	.049
WASO	Non-literal speech 1	32.6 (7)	.986	.143	.023
	Non-literal speech 2	44.7 (7)	.982	.173	.029
	Verbal Metaphors in Context	36.4 (7)	.981	.168	.020
	Nominal Metaphors in Context	43.1 (7)	.980	.170	.025
	Proverbs in Context	26.4 (7)	.990	.138	.021
	Third order ToM stories	39.4 (7)	.983	.176	.028
	TASIT-PART 1 (Neutral)	19.0 (7)	.991	.110	.015
	TASIT-PART 1 (Happy)	17.3 (7)	.993	.091	.016
	TASIT-PART 1 (Decoding of uncertain emotional expressions)	24.5 (7)	.991	.118	018
TST	Nominal Metaphors in Context	33.2 (7)	.982	.144	.055
	Proverbs in Context	32.3 (7)	.985	.142	.050
	Third order ToM stories	45.0 (7)	.976	.174	.072
	TASIT-PART 1 (Neutral)	14.1(7)	.994	.075	.039
	TASIT-PART 1 (Happy)	16.0 (7)	.991	.085	.038
	TASIT-PART 1 (Decoding of uncertain emotional expressions)	23.1 (7)	.990	.113	0.51

Note.1. *χ^2 = chi-square test statistic; df = degrees of freedom; CFI = Comparative Fit Index; RMSEA = Root Mean Square Error of Approximation; SRMR = Standardized Root Mean Square Residual.*
2. *The TST models for Non-literal Speech 1, Non-literal Speech 2, and Verbal Metaphors in Context are presented in the main manuscript and are therefore omitted from this supplementary table.*

Table S9. Standardized prospective cross-lagged path coefficients for the longitudinal associations between objective sleep parameters and ToM outcomes.

**Sleep Parameter**	**Outcome**	**Path**	**β**	**SE**	**95% CI**	**p**
SE	Non-literal speech 1	T1→T2	.035	.003	[−.002, .009]	.81
		**T2→T3**	**.108**	**.005**	**[.004, .025]**	**.006**
	Non-literal speech 2	T1→T2	.005	.006	[−.010, .014]	.756
		T2→T3	.041	.013	[−.011, .040]	.269
	**Verbal Metaphors in Context**	**T1→T2**	**−.039**	**.003**	**[−.010, .003]**	**.051**
		T2→T3	.093	.004	[−.000, .016]	.062
	Nominal Metaphors in Context	T1→T2	−.022	.003	[−.008, .004]	.458
		T2→T3	.039	.004	[−.004, .012]	.334
	Proverbs in Context	T1→T2	.020	.004	[−.004, .011]	.392
		T2→T3	−.013	.005	[−.011, .007]	.624
	Third order ToM stories	T1→T2	−.001	.002	[−.004, .004]	.951
		T2→T3	.040	.004	[−.003, .011]	.277

	TASIT-PART 1 (Neutral)	T1→T2	.067	.003	[−.001, .009]	.154
		T2→T3	.075	.003	[−.002, .011]	.203
	TASIT-PART 1 (Happy)	T1→T2	.063	.004	[−.003, .012]	.288
		T2→T3	.025	.005	[−.008, .012]	.687
	TASIT-PART 1 (Decoding of uncertain emotional expressions)	T1→T2	.025	.002	[−.002, .007]	.233
		T2→T3	.019	.004	[−.006, .010]	.577
WASO	Non-literal speech 1	T1→T2	−.004	.001	[−.001, .001]	.837
		T2→T3	−.056	.001	[−.003, .001]	.152
	Non-literal speech 2	T1→T2	.013	.001	[−.001, .003]	.435
		T2→T3	.003	.003	[−.005, .005]	.936
	Verbal Metaphors in Context	T1→T2	.044	.001	[−.001, .002]	.399
		T2→T3	−.08	.001	[−.003, .000]	.102
	Nominal Metaphors in Context	T1→T2	.044	.001	[−.000, .002]	.131
		T2→T3	−.041	.001	[−.002, .001]	.312
	Proverbs in Context	T1→T2	−.02	.001	[−.002, .001]	.389
		T2→T3	.03	.001	[−.001, .003]	.321
	Third order ToM stories	T1→T2	−.003	.000	[−.001, .001]	.893
		T2→T3	−.003	.001	[−.001, .001]	.938
	TASIT-PART 1 (Neutral)	T1→T2	.001	.000	[−.001, .001]	.956
		T2→T3	.009	.001	[−.002, .001]	.797
	TASIT-PART 1 (Happy)	T1→T2	−.013	.001	[−.002, .001]	.824
		T2→T3	−.16	.001	[−.002, .002]	.797
	TASIT-PART 1 (Decoding of uncertain emotional expressions)	T1→T2	.001	.000	[−.001, .001]	.959
		T2→T3	.009	.001	[−.001, .002]	.797
TST	Nominal Metaphors in Context	T1→T2	.038	.000	[−.000, .001]	.188
		T2→T3	.055	.001	[−.000, .002]	.168
	Proverbs in Context	T1→T2	−.013	.001	[−.001, .001]	.605
		**T2→T3**	**.067**	**.001**	**[.000, .003]**	**.013**
	Third order ToM stories	T1→T2	−.006	.000	[−.001, .001]	.769
		**T2→T3**	**.079**	**.000**	**[.000, .002]**	**.030**
	TASIT-PART 1 (Neutral)	T1→T2	.068	.000	[−.000, .001]	.149
		T2→T3	.054	.000	[−.000, .001]	.367
	TASIT-PART 1 (Happy)	**T1→T2**	**.172**	**.000**	**[.001, .002]**	**.003**
		**T2→T3**	**.099**	**.001**	**[−.000, .002]**	**.115**
	TASIT-PART 1 (Decoding of uncertain emotional expressions)	T1→T2	.052	.000	[.000, .001]	.013
		**T2→T3**	**.100**	**.001**	**[.000, .002]**	**.003**

Note 1. *Only the prospective cross-lagged paths from sleep parameters to social cognition outcomes are presented. Autoregressive paths, residual covariances, and indirect effects are omitted for brevity. Standardized coefficients (β), standard errors (SE), 95% confidence intervals (CI), and p values are reported.*

2. *The TST models for Non-literal Speech 1, Non-literal Speech 2, and Verbal Metaphors in Context are presented in the main manuscript and are therefore omitted from this supplementary table.*

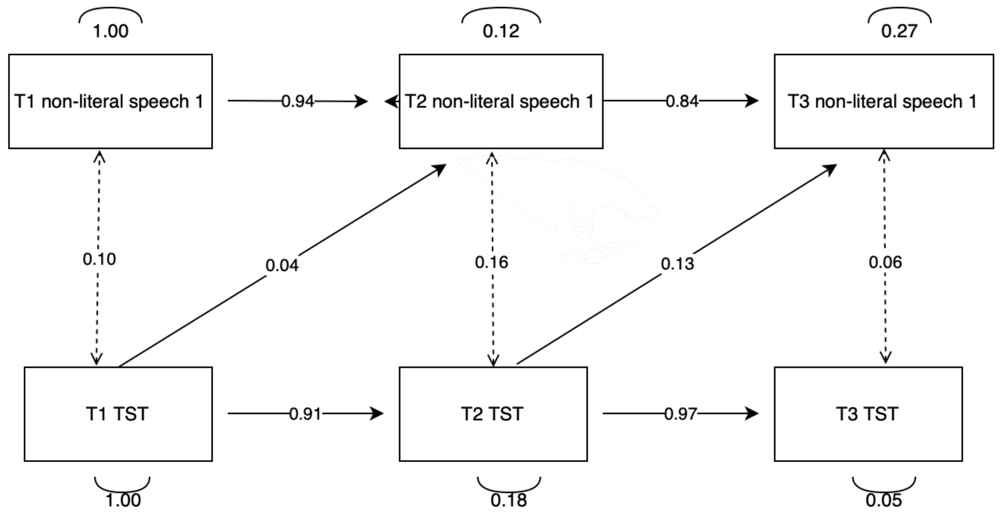

Figure S1. Longitudinal path model illustrating the associations between TST and non-literal speech 1 across the three assessment waves.

Note. Standardized path coefficients (β) are presented. The complete model structure, including non-significant paths, is displayed.

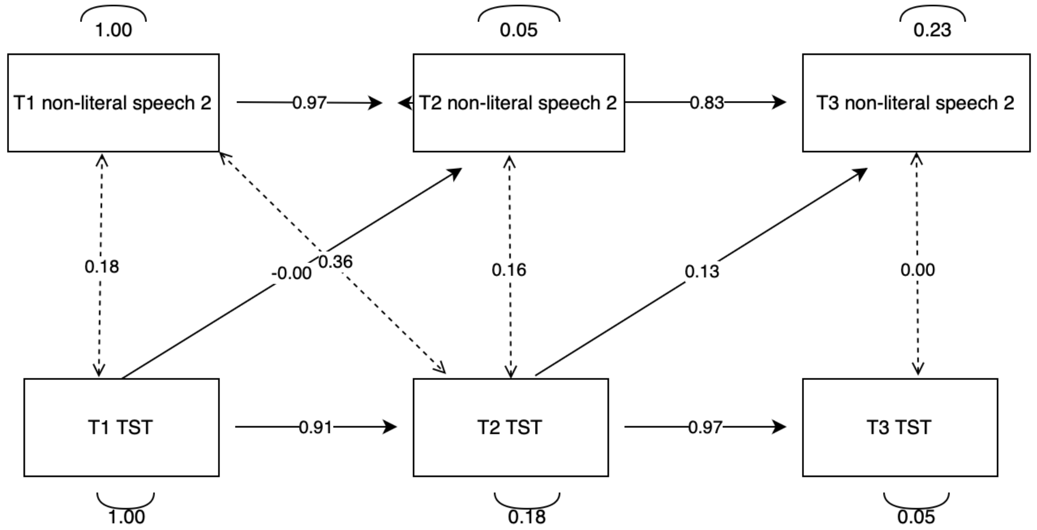

Figure S2. Longitudinal path model illustrating the associations between TST and non-literal speech 2 across the three assessment waves.

Note. Standardized path coefficients (β) are presented. The complete model structure, including non-significant paths, is displayed.

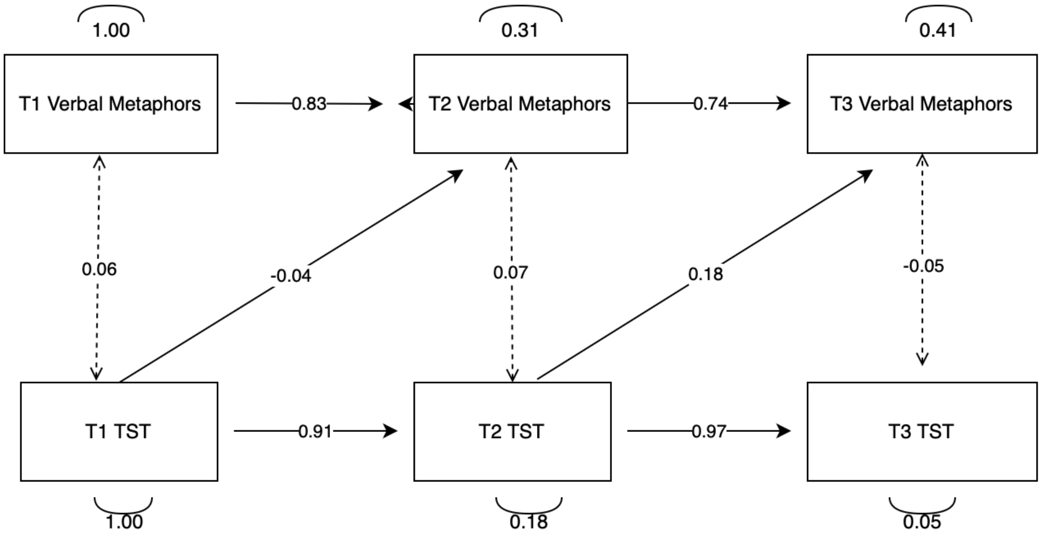

Figure S3. Longitudinal path model illustrating the associations between TST and Verbal Metaphors in context across the three assessment waves.

Note. Standardized path coefficients (β) are presented. The complete model structure, including non-significant paths, is displayed.
